# Recent Insights into Protein-Polyphenol Complexes: Molecular Mechanisms, Processing Technologies, Synergistic Bioactivities, and Food Applications

**DOI:** 10.3390/molecules31020287

**Published:** 2026-01-13

**Authors:** Hoang Duy Huynh, Thanh Huong Tran Thi, Thanh Xuan Tran Thi, Parushi Nargotra, Hui-Min David Wang, Yung-Chuan Liu, Chia-Hung Kuo

**Affiliations:** 1 Department of Seafood Science, National Kaohsiung University of Science and Technology, Kaohsiung 81157, Taiwan; i113199116@nkust.edu.tw; 2 Institute of Aquatic Science and Technology, National Kaohsiung University of Science and Technology, Kaohsiung 81157, Taiwan; 3 Faculty of Applied Technology, Yersin University of Dalat, Dalat 670000, Vietnam; huongttt@yersin.edu.vn (T.H.T.T.); xuanttt@yersin.edu.vn (T.X.T.T.); 4 Department of Chemical Engineering, National Chung Hsing University, Taichung 40227, Taiwan; 5 Graduate Institute of Biomedical Engineering, National Chung Hsing University, Taichung 40227, Taiwan; 6 Center for Aquatic Products Inspection Service, National Kaohsiung University of Science and Technology, Kaohsiung 81157, Taiwan

**Keywords:** protein-polyphenol complexes, bioactive compounds, molecular mechanisms, synergistic effects, preservation

## Abstract

Modifying proteins through grafting with polyphenols has received much attention recently due to its immense application potential. This stems from the formation of protein-polyphenol complexes, altering the structural and functional properties of the constituent molecules. In food systems, the interaction between proteins and polyphenols, including covalent and non-covalent binding, represents a green, simple, and effective strategy to transform difficult-to-process protein sources into high-value functional ingredients. In addition, the complexes formed can increase stability, biological activity, and bioavailability of polyphenols, thereby expanding their applications. Gaining insight into protein-polyphenol complexes is essential for developing novel complexes, formulations, and other applications utilizing protein and natural polyphenols. Thus, this review outlines the binding affinities and interaction mechanisms, explains factors affecting complex formation, revisits structural modulation of protein, modern processing technologies, and systematically discusses the synergistic bioactivities of the resulting complexes. We also discuss strategies to address the applications of protein–polyphenol complexes for developing functional food products with prolonged shelf life. These applications can be expanded to other industrial areas, such as pharmaceuticals and material engineering, contributing towards better nutritional quality, beneficial healthy aspects, and sustainability.

## 1. Introduction

Recently, more attention has been directed towards healthier and superior protein-based alternatives because consumers seek sustainable and healthy alternatives to animal-based proteins [1]. Structurally, proteins have unique interface properties for the hydrophilic and hydrophobic regions [2]. Thanks to the diverse hydrophobic-lipophilic nature of proteins, coming from structural differences, protein reflects good biocompatibility. Functionally, food proteins, such as myofibrillar protein [3], zein [4], soy protein isolate [5], and pea protein isolate [6], exhibit excellent surface properties and self-assembly capabilities. They also show strong emulsifying and gelling properties. Thus, they can be used as a general carrier for micronutrient transport [7]. However, the use of protein is often limited in the food industry. This is because, under processing, storage, or consumption conditions, many factors, such as changes in pH, ionic strength, and temperature, can lead to changes in its structure. These structural changes adversely affect its functionalities and biological activity [8]. To broaden their applications, their abilities can be improved by modifying the protein structure [9].

In nature, many of the most important biological functions involve polyphenols. These are secondary plant metabolites containing various complex structures with aromatic benzenoid rings and hydroxy groups [10]. With diverse structures from single molecules to complex high-molecular-mass polymers, polyphenols also exhibit diverse physiological properties. These include antibacterial, anti-cancer, cardiovascular disease-preventive, anti-oxidative, and other health-related and functional properties [11]. However, some polyphenols, such as flavonoids, catechins, and tannins, are severely limited in their application in food formulations due to their poor water dispersibility and chemical stability [12]. In addition, the complexity of the food matrices can influence the bioavailability of phenolic compounds. During digestion, polyphenols can undergo various interactions, which alter their accessibility [13]. To overcome these disadvantages, the conjugation between protein and polyphenol seems to be an effective approach in property improvements of certain food products. The complexes formed by polyphenols and proteins can increase stability, biological activity, and polyphenols’ bioavailability, which contribute to expanding their application in functional foods.

Through grafting, conjugation or polymerization, molecular modification offers a promising method for synthesizing novel materials or modifying biomolecules to achieve their desired physical and chemical properties [14]. Such an approach is particularly useful for phenolic-based biopolymers, where protein-polyphenol complexes have been shown to possess better functional properties and stability than single proteins [15]. The interactions between proteins and polyphenols may be either covalent or non-covalent [12]. Tentative identifications indicate that single polyphenol molecules can bond to one or two amino acids, and polyphenol dimers may link to one or two residues. Thus, this illustrates the complexity of covalent protein–polyphenol conjugation [16]. From the Michael addition and Schiff base reactions, the covalent interaction yields a variety of products, such as monomeric, dimeric, and polymeric protein-polyphenol complexes. Specific adducts are protein-S-polyphenol and protein-N-polyphenol complexes [17,18]. Non-covalent forces, including hydrogen bonding, hydrophobic interactions, electrostatic attraction, van der Waals forces, and π–π stacking, often act simultaneously to form complex protein–polyphenol complexes [14]. Non-covalent interaction is the most abundant in nature and is susceptible to changes in environmental factors, such as pH and temperature. The other factors that influence the formation of protein-polyphenol complexes are different types of polyphenol compounds [19], polyphenol-to-protein ratio [15], ionic strength [20], and molecular size [21]. Therefore, understanding these interactions is essential for the development of novel complexes that exhibit enhanced functional properties and bioactivities for various applications.

Numerous strategies have been investigated to facilitate the formation of covalent bonds between polyphenols and proteins. These include enzymatic approaches, non-enzymatic methods such as alkaline [22] and free radical grafting [3], chemical coupling techniques [23], and contemporary physical methods like ultrasound [17] and cold plasma [24]. The alkaline method offers a simple and effective way to produce polyphenol-protein complexes [4]. With this strategy, it becomes possible to enhance the functionality, bioavailability, and biological activities of the resulting complexes. These enhancements are related to antioxidant, anti-inflammatory, enzyme inhibitory, anticancer, antitumor, antiviral, and antibacterial properties [12,25,26,27,28].

In recent years, many practical studies have investigated complexes of plant-based proteins and polyphenols. These studies focus on how techno-functional properties and health benefits are improved, such as antioxidant activity or reduced allergenicity [29,30]. To our knowledge, the existing literature has largely focused on narrow segments, such as a specific type of protein like whey protein or a particular type of interaction, such as mainly non-covalent interactions [8,10,31]. Therefore, unlike previous works limited to specific interactions, this review establishes a comprehensive framework that integrates diverse research domains. Here, we present a novel perspective on molecular interaction mechanisms and their synergistic bioactivities in protein-polyphenol complexes. We discuss viewpoints on advanced processing technologies and their proposed applications in food preservation and functional foods. Finally, we outline recent challenges and future perspectives that demonstrate the potential of these complexes as attractive delivery platforms for commercially feasible approaches. The insights presented here will be beneficial to academic and industrial researchers who aim to formulate or utilize protein and natural polyphenols in food systems.

## 2. Molecular Protein-Polyphenol Interaction Mechanisms

### 2.1. Covalent Bonding

Nucleophilic enrichment of stable covalent bonds between polyphenols and proteins is an essential process, typically initiated by the oxidation of polyphenols, followed by nucleophilic attack from protein residues. This results in irreversible interactions that improve the physicochemical properties and stability of proteins, rendering the resulting complexes suitable for diverse applications, particularly in the food industry [12,17]. In the initial phase, polyphenols are oxidized to produce highly reactive intermediates, specifically quinones or semi-quinones, through various mechanisms. Enzymes, including polyphenol oxidases (PPO), laccase, and tyrosinase, catalyze the transformation of monophenols into *o*-diphenols and then into o-quinones in the presence of oxygen [32]. Alkaline conditions (e.g., pH 9.0) facilitate the spontaneous oxidation and reconfiguration of polyphenols into quinones [17,33]. Additionally, free radicals from initiator systems, such as ascorbic acid/hydrogen peroxide redox pairs or ultrasound, can oxidize polyphenols [17,33]. Hydrogen peroxide and *o*-quinones are generated by autoxidation processes, which are influenced by the structure, concentration, temperature, and pH of polyphenols [16]. In addition, polyphenols can undergo polyphenol autoxidation, which is the process by which transition metal ions such as Fe^2+^/Fe^3+^ and Cu^+^ accelerate the oxidation of polyphenols to quinones. These quinones are electrophilic intermediates that can be oxidized again to make aggregates with a greater molecular weight and quickly react with nucleophilic groups on proteins [34].

In the second stage, reactive quinones engage with nucleophilic sites on proteins using two main mechanisms: Michael addition and Schiff base creation. This reaction produces irreversible covalent adducts [16]. The principal nucleophilic targets are cysteine’s sulfhydryl/thiol (-SH) and lysine’s amino (-NH_2_) groups [17,35,36]. However, other residues, including proline, histidine, tryptophan, methionine, tyrosine, and arginine, can also be involved [35,37]. In general, thiol groups react at a rate of approximately 10^5^ times that of amine groups [16,20]. However, the reaction of 4-methylbenzoquinone with β-lactoglobulin can occasionally result in a more extensive modification of lysine residues, as evidenced by the protein structure and lysine abundance [36].

The Michael addition mechanism involves the direct addition of nucleophilic amino acid side chains, such as the thiol group of cysteine or the amino group of lysine, to electron-deficient carbon atoms of the *o*- or *p*-quinone structure. Consequently, this reaction forms stable C–S bonds for cysteine or C–N bonds for lysine and other amino groups, establishing strong covalent links between polyphenols and proteins [3,6].

The Schiff base mechanism, on the other hand, is a condensation reaction between the carbonyl groups of quinones (aldehyde or ketone) and primary amines on proteins, usually the ε-amino group of lysine. This reaction progresses by eliminating a water molecule, which leads to the formation of an imine (C=N) bond. This bond is responsible for the C–N linkage in protein–polyphenol complexes [16,38]. The formation of Schiff base adducts is favored at elevated temperatures, such as approximately 120 °C, which facilitates the condensation reaction [16]. For example, in the reaction between 4-methylcatechol (4MC) and β-lactoglobulin (β-LG), 4MC under oxidative conditions, such as alkaline pH (e.g., pH 8.0) and aerobic exposure, transforms into 4-methylbenzoquinone (4MBQ), a highly electrophilic o-quinone [16]. Consequently, this 4MBQ nucleophilically assaults β-LG at reactive residues, primarily Cys, Lys, Arg, His, and Trp. This reaction is primarily mediated by the Michael addition mechanism, which results in the formation of C–S or C–N bonds at sites such as Cys-121 (identified as the most likely modified residue), Lys-8, Lys-14, and Trp-19. Formation of Schiff bases is also seen on residues like Lys-91 and, to a lesser extent, Lys-14. The final product is 4MBQ-modified β-lactoglobulin (β-LQ) [34,35].

**Figure 1:**
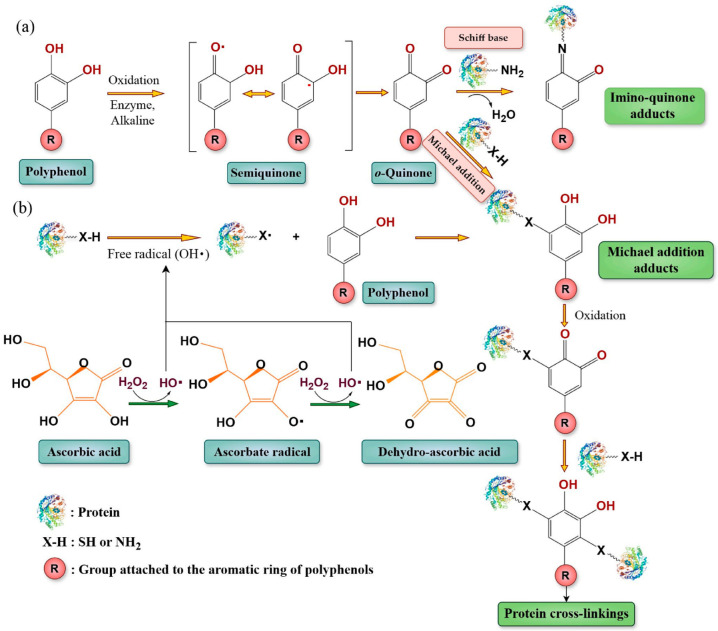
Covalent bond formation pathways between polyphenols and proteins via alkaline, (**a**) enzymatic and alkaline oxidation leading to Schiff base formation and Michael addition, and (**b**) the free radical coupling method.

Overall, the covalent interactions through both Michael addition and Schiff base reactions yield a variety of products, including monomeric, dimeric, and polymeric protein–polyphenol complexes [35,38]. Specific adducts include quinone-thiol (protein-S-polyphenol) and quinone-amine (protein-N-polyphenol) complexes [17,18]. For instance, EGCG reacts with β-lactoglobulin to form monomeric (18.3 kDa) and dimeric (37.6 kDa) complexes [13], while curcumin reacts with soy protein isolate under alkaline conditions to form stable hydroxyquinone dimers [39]. Tentative identifications further indicate that single polyphenol molecules can bond to one or two amino acids, and polyphenol dimers may link to one or two residues, thus illustrating the complexity of covalent protein–polyphenol conjugation [16]. The mechanism of covalent bond formation between polyphenols and proteins is illustrated in Figure 1.

### 2.2. Non-Covalent Bonding

**Figure 2:**
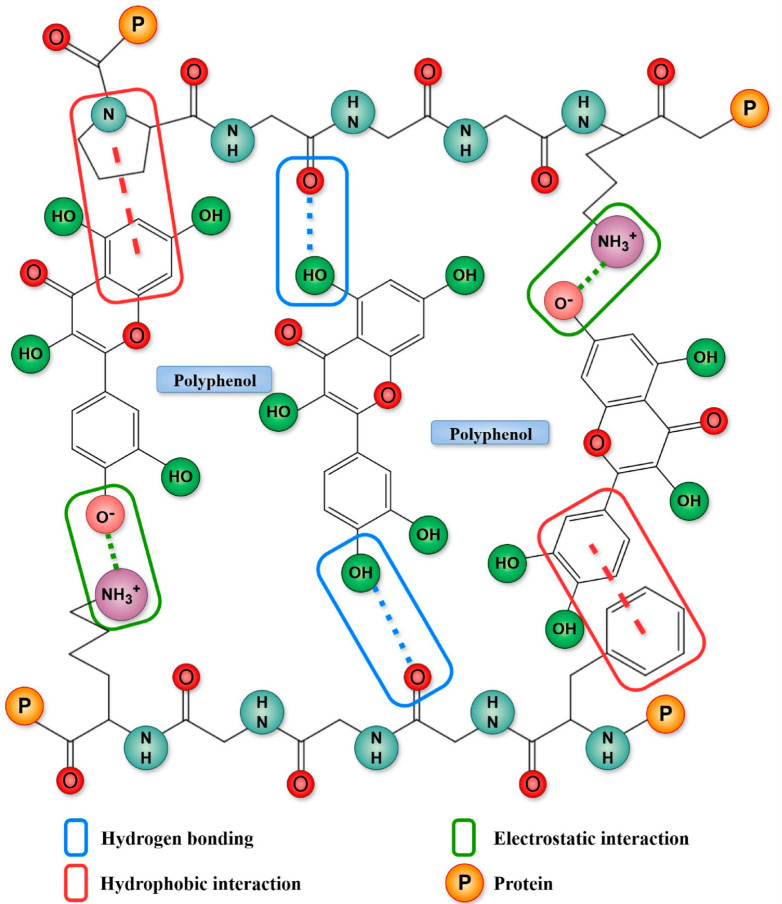
Mechanism of non-covalent bonding between polyphenols and proteins.

Polyphenols and proteins engage in non-covalent interactions defined by weak, reversible, and low-energy bonds, which occur naturally under mild conditions, including physiological pH and temperature. Because of this, they provide a safe and effective way to change how proteins work and make food better. Understanding these interactions is essential for the development of novel complexes that exhibit enhanced functional properties and bioactivities for various applications. Non-covalent forces include hydrogen bonding, hydrophobic interactions, electrostatic attraction (Figure 2), van der Waals forces, and π–π stacking, which often act simultaneously to form complex protein–polyphenol complexes [14].

Hydrogen bonding is a fundamental interaction because the hydroxyl groups in polyphenols are very good at donating hydrogen. These hydroxyl groups can easily form strong hydrogen bonds with the carboxyl (C=O), amino (-NH), and hydroxyl (-OH) groups in the protein’s backbone and side chains [14]. This is especially true for amino acid residues that are polar, such as threonine, tyrosine, serine, and asparagine [40]. The formation of hydrogen bonds is essential for improving the stability and selectivity of polyphenol-protein complexes [41]. Wang et al. (2023) [42] verified that the non-covalent interaction of soybean isoflavones (SI) with whey protein isolate (WPI) or succinylated WPI (SAn-WPI) predominantly transpires via hydrogen bonding, engaging specific residues such as Asn109, Ser116, Lys69, Ile56, and Lys70 of β-Lg, along with Tyr103, Lys108, Ile33, His32, and Phe31 of α-La. Zhang et al. (2025) [43] showed that the coffee flavonoids luteolin (LUT) and quercetin (QC) mostly interact with β-casein (β-CN) via hydrogen bonds and van der Waals forces. Molecular docking showed that luteolin made three hydrogen bonds with β-CN at LYS105, TYP109, and PRO116. Quercetin made three hydrogen bonds with β-CN at GLN152, THR107, and PRO116.

Hydrophobic interactions occur due to the linkage of the non-polar aromatic rings of polyphenols with the hydrophobic amino acid residues of proteins (such as leucine, isoleucine, glycine, methionine, alanine, phenylalanine, valine, tyrosine, cysteine, and tryptophan) [14]. This connection reduces the contact between hydrophobic molecules and the aqueous environment, which stabilizes the protein-polyphenol complex and often lowers the protein’s surface hydrophobicity, making it easier to dissolve [43]. Liu et al. (2024) [21] reported that the interactions for wheat germ protein (WGP) with piceatannol (PIC) and ferulic acid (FA) complexes were mainly hydrophobic interactions. Zhang et al. (2025) [43] observed that apigenin (AG) and EGCG mostly interact with β-casein (β-CN) via hydrophobic interactions. Molecular docking revealed that AG formed seven hydrophobic bonds (e.g., PRO102, PRO154, LEU136), and EGCG formed five hydrophobic contacts (e.g., TYR109, PRO154) with specific proline and tyrosine residues on β-CN. Nimaming et al. (2024) [44] found that potato protein (PoP) binds to quercetin (QC) mostly through hydrophobic interactions, where the aromatic amino acids of PoP connect with the phenolic rings of QC.

Electrostatic interactions, also known as ionic bonds, transpire between charged groups on proteins and polyphenols [21]. This often entails the interaction between the positively charged protein groups, such as the *ε*-amino groups of lysine, and the negatively charged hydroxyl groups on polyphenols [14]. Although typically subordinate to hydrogen bonding and hydrophobic interactions, these connections enhance the overall stability of the complex [21]. A clear illustration of this is provided by Liu et al. (2024) [21], who identified electrostatic interaction as a main driving force for the formation of complexes between wheat germ protein (WGP) and chlorogenic acid (CA). Li et al. (2024) [30] reported that Asp48 and Asp52 bind to theaflavin (TF) via electrostatic forces, and TF interacts with Lys13, Asp18, Asp48, and Asp52 through electrostatic forces with lysozyme.

Van der Waals forces are weak, short-range intermolecular forces that include dipole–dipole interactions, dipole-induced dipole interactions, and London dispersion forces, and these forces arise from temporary fluctuations in electron distribution around atoms, creating transient dipoles that induce complementary dipoles in neighboring molecules [21,45]. Zhang et al. (2025) [43] stated that luteolin (LUT) and quercetin (QC) primarily interacted with β-casein (β-CN) through hydrogen bonds and van der Waals forces. Additionally, apigenin (AG) was encircled by nine van der Waals forces, including LEU146 on β-CN, while EGCG was encompassed by 13 van der Waals forces, including VAL108. Another instance of this relationship is that Athapaththu et al. (2023) [46] observed many van der Waals interactions with surrounding amino acids when pinostrobin bound to tyrosinase.

π–π stacking interactions are a form of non-covalent attraction that occurs when the electron clouds of aromatic rings align in a stabilizing manner. In protein–polyphenol systems, interactions generally include the aromatic rings of polyphenols and aromatic amino acid residues, including tryptophan, tyrosine, and phenylalanine. The rings can have geometries such as parallel-displaced or T-shaped, which facilitate advantageous stability. In metal–polyphenol networks (MPNs), π–π stacking typically transpires among polyphenol molecules, but non-covalent coordination with metal ions like Fe^3+^ is predominantly reinforced by hydrogen bonding rather than π–π stacking. Computational analyses suggest that the hydroxyl group on the pyran ring can promote T-shaped π–π stacking, facilitating this interaction in epigallocatechin (EGC) and procyanidin (PC), but not in catechin (CAT), which does not possess a conducive geometry [47].

### 2.3. Computational Analysis of Protein-Polyphenol Interactions

#### 2.3.1. Molecular Docking

Molecular docking is a computer method that tries to guess how tiny ligands like polyphenols will bind to protein receptors and how they will interact with one another at the molecular or atomic level [39,48]. In virtual screening, it is used a lot to find the best binding modes and active sites. It also uses scoring methods to estimate binding strength, where lower (more negative) binding energies mean that the complexes are stable and favorable [38,49]. This method also shows the main forces that keep the interaction stable, such as hydrogen bonding, hydrophobic contacts, van der Waals forces, and electrostatic interactions. It often names the amino acid residues that are involved [38,50]. For instance, when lactoferrin (LF) and chlorogenic acid (CGA) were docked together, a binding cavity was found at the intersection of LF’s N and C leaves. This cavity was held together by hydrophobic contacts, van der Waals forces, and hydrogen bonding [38,51]. The docking procedure usually starts with constructing the receptor and ligand structures, which are often taken from the Protein Data Bank and PubChem. Then, blind or site-specific docking is performed to find possible binding sites [49,52]. The resulting binding energies, typically denoted in kcal/mol, offer a quantitative assessment of affinity [42,49]; for example, the docking of apigenin with blood–brain barrier (BBB)–associated proteins produced binding scores ranging from −5.1 to −10.13 kcal/mol, with Matrix Metalloproteinase-9 (MMP-9) anticipated to exhibit the most robust binding [49].

#### 2.3.2. Molecular Dynamics Simulations

In addition to docking, molecular dynamics (MD) simulations are used to test the stability and time-dependent behavior of polyphenol–protein complexes. This gives a more realistic view of the atomic level [53,54]. By tracking conformational changes and ligand flexibility, MD validates docking predictions and reveals the dynamic persistence of hydrogen bonds and hydrophobic contacts across the simulation period, often extending to 100 ns or longer [54,55]. Several parameters are used to assess these complexes, including RMSD, which monitors structural deviation and stability; RMSF, which identifies flexible amino acid residues (interaction between β); SASA, which reflects protein compactness; and interaction energy profiles that confirm electrostatic and van der Waals contributions. Case in point, MD investigations on β-lactoglobulin (BLG) demonstrated that apigenin decreased RMSD values and SASA, signifying improved stability and compactness, whereas eriodictyol yielded steady fluctuations that surpassed those of luteolin [56]. Additionally, sophisticated methodologies like MM/GBSA enhance binding free energy (ΔG–bind) calculations derived from molecular dynamics (MD) trajectories, yielding reliable affinity predictions [53,57]. One study showed that polyphenol–peptide complexes like gallate–TNYLFSPNGPIA with Ephrin receptors had very negative ΔG–bind values. The strongest binding was seen for coumarate–TNYLFSPNGPIA with ATP-bound EphB2 (−167.11 kcal/mol), which shows that conjugation can make receptor interactions much stronger [57].

## 3. Factors Influencing Complex Formation

### 3.1. Structural Characteristics of Polyphenols

In covalent interactions, polyphenols with elevated hydroxyl content are more prone to oxidizing into quinones. This enables the formation of covalent connections with nucleophilic residues, hence increasing the frequency of grafting and enhancing the functioning of the molecules [24,34]. For example, the number of hydroxyl groups determines the strength of covalent interactions with proteins such as ovalbumin (OVA) [19]. On the other hand, cyanidin-3-O-glucoside (C3GQ), which has a lot of hydroxyl groups, has much better antioxidant activity [34]. However, an overabundance of hydroxyl groups can disrupt protein hydrogen bonding and compromise structural integrity [58]. Beyond the quantity of hydroxyl groups, their position is equally important. Polyphenols containing catechol (two adjacent –OH) or galloyl (three adjacent –OH) groups are readily oxidized to *o*-quinones, which strongly drive covalent attachment [16,34]. In particular, the catechol moiety located on the B ring of flavonoids is more reactive than the same group on the A ring [16]. Additionally, the configuration and quantity of aromatic rings in polyphenols significantly influence their covalent interactions with proteins. The essential carbon structure—such as C6–C3–C6 in flavonoids, C6–C3 in hydroxycinnamic acid derivatives, or C6–C1 in hydroxybenzoic acid derivatives—profoundly influences binding behavior [36]. More rings, such as the C-ring in flavonoids, can alter autoxidation and produce more H_2_O_2_, which facilitates the formation of covalent bonds [16].

In non-covalent interactions, the number and position of hydroxyl groups are critical in defining hydrogen bonding capacity, hydrophilicity, and protein affinity [21,59]. For instance, EGCG, with eight hydroxyl groups, shows stronger binding to wheat germ protein (WGP) than chlorogenic acid, piceatannol, or ferulic acid, which contain fewer hydroxyl groups [21]. Nevertheless, excessive hydroxyl participation in hydrogen bonds can sometimes mask antioxidant activity, as observed in resveratrol–BSA complexes [30,60]. Structural motifs are equally important: catechol and galloyl moieties act as major contributors to hydrogen bonding [47], while hydroxyl groups in anthocyanins serve as principal interaction sites [15]. In catechin, ortho-phenolic groups on the B or C rings and hydroxyls on the A ring make it better at scavenging free radicals and protect myofibrillar proteins from oxidation [58]. The pyran-ring hydroxyl in epigallocatechin (EGC) also makes very strong hydrogen bonds since it has a high electronegativity [47]. The arrangement of aromatic rings and their substituents, along with hydroxyl groups, affects non-covalent binding. Aromatic rings facilitate hydrophobic interactions and π–π stacking. Substituents can either make or break the affinity by changing the electron density or causing steric hindrance [15,47]. For instance, the methoxyl groups of ferulic acid and sinapic acid make it harder for BSA to attach to them than chlorogenic and caffeic acids. Gallic acid mostly operates through electrostatic interactions [21], while EGCG engages with BSA via hydrogen bonds and π–π stacking. Trans-resveratrol binds better to BSA in a less watery environment than β-lactoglobulin or α-lactalbumin. This suggests that being hydrophobic makes binding stronger. In addition to these structural characteristics, dynamic aspects such as molecular flexibility and isomerism can affect the functionality of non-covalent interactions. To illustrate, cis-resveratrol binds to BSA more strongly than it does to milk proteins [60]. These interactions often cause proteins to change shape. For example, when wheat germ protein unfolds, tryptophan residues are exposed to polar environments, which makes the fluorescence less intense [21]. However, excessive binding among high concentrations of polyphenols might lead to abnormal aggregation. For instance, catechin makes soy protein isolate stick together and makes it less soluble [12]. In contrast, tea polyphenols make myofibrillar proteins form uneven particles [58]. After exploring the role of polyphenol functional groups in shaping protein–polyphenol complexes, we now turn to other key factors that govern the formation and stability of these complexes.

### 3.2. Polyphenol-to-Protein Ratio

The ratio of polyphenols to protein is a critical determinant of both interaction mechanisms and the properties of resulting complexes [15,61]. At lower ratios, polyphenols often act as multi-site ligands in a multidentate process, binding to several protein molecules or sites and thereby promoting crosslinking and the formation of dimers or oligomers, particularly with larger polyphenols. In contrast, significantly elevated polyphenol concentrations are necessary for the monodentate process, when several polyphenol molecules attach to a single protein molecule [15].

This ratio also influences protein structure and function. In low concentrations, phenolic compounds can partially disrupt the tertiary structure of proteins while preserving the α-helix content. This makes the surface of complexes more hydrophobic and less soluble [61]. Consistently, Hu et al. (2022) [62] found that the degree of myofibrillar protein aggregation correlated with polyphenol levels, highlighting ratio-dependent effects on protein assembly.

The antioxidant capacity of polyphenol–protein complexes is similarly influenced. In general, increasing polyphenol concentrations enhances antioxidant activity (Molecular Mechanisms). Thongzai et al. (2022) [63] conducted research that showed the antioxidant activity of whey protein-phenolic complexes rose as the concentration of phenolic acid increased from 0.5% to 5%. Wang et al. (2021) [64] have similarly shown that covalent connections between high hydrostatic pressure-pretreated rice bran protein hydrolysates and ferulic acid at elevated concentrations enhanced antioxidant capabilities.

However, high quantities can be harmful. High polyphenol levels can affect protein structure and reduce the antioxidant capabilities of complexes [32]. To illustrate, at 500–1000 mg L^−1^, EGCG inhibited myofibrillar protein emulsification and gel formation [16]. Yet, low doses of oxidized EGCG produced a stable crosslinked network with soy protein isolate. But at greater concentrations, reactive protein sites were saturated, which prevented effective crosslinking [61].

### 3.3. Ionic Strength

Ionic strength, mostly determined by salt concentration, significantly influences protein–phenolic interactions by affecting electrostatic forces and binding affinity [20]. In particular, depending on the type of non-covalent interactions involved, ionic strength can either enhance or weaken binding. This is because the effect is often associated with charge neutralization, which may consequently facilitate or hinder the formation of complexes between positively charged protein groups and negatively charged phenolic groups [20]. For instance, the interaction of tannic acid with proteins or other macromolecules is strongly dependent on the surrounding medium, particularly ionic strength and pH [65]. Beyond binding affinity, ionic strength also impacts the assembly and stability of biopolymer nanocomplexes, together with other factors such as pH, protein concentration, polysaccharide density, and macromolecular composition [59]. In line with this, the oxidative stability of myofibrillar protein–EGCG complexes was found to improve after storage, centrifugation, pH adjustment, and salt treatment, suggesting that salt concentration is an important factor affecting their stability [16].

### 3.4. Molecular Size

The molecular size or molecular weight of polyphenols is a key determinant of interaction mechanisms, binding affinity, and the structural and functional outcomes of polyphenol–protein complexes. Generally, higher molecular weight polyphenols demonstrate a greater affinity due to their bigger structures providing many binding sites. J. Liu et al. (2024) [21] indicated that EGCG had a stronger binding affinity to wheat germ protein compared to smaller molecules like chlorogenic acid, piceatannol, and ferulic acid. In a similar vein, Li et al. (2023) [47] demonstrated that procyanidin, the largest flavonoid examined, produced the thickest and most effective coatings in metal–polyphenol networks in contrast to smaller flavonoids such as epigallocatechin and catechin. Bayati and Poojary (2025) [16] also emphasized that molecular weight substantially influences the stability and characteristics of polyphenol–protein interactions.

Large polyphenols, such as tannins and proanthocyanidins, function as multidentate ligands, facilitating concurrent interactions with multiple protein sites, which enhances crosslinking or structural stabilization. Krekora et al. (2022) [66] stated that tannic acid kept the gluten network stable while the dough was being mixed, whereas smaller gallic and ellagic acids broke it up. Moreover, a higher degree of polymerization of procyanidin strengthened its interaction with cell wall materials, highlighting the role of multivalent binding [15].

In addition, polyphenol size influences aggregation and particle formation, as Dai et al. (2023) [12] reported that elevated catechin concentrations led to the formation of large insoluble aggregates with soy protein isolate. Xuepeng Li et al. (2020) [3] found that excessive tea polyphenols triggered oxidative denaturation and irregular aggregation of myofibrillar proteins, thereby enlarging particle size. Du et al. (2024) [67] further showed that conjugation of *Pseudosciaena crocea roe* protein isolate with EGCG increased protein molecular weight from 86.9 to 215.3 kDa.

Finally, size also modulates functional outcomes. For instance, Chen et al. (2020) [68] showed that the antioxidant potential of whey protein isolate–EGCG complexes was higher than that of whey protein isolate–quercetin. Dai et al. (2023) [12] also found that covalent complexes of soy protein isolate with catechin had more antioxidant activity than their non-covalent counterparts. This indicates that covalent crosslinking enhanced their stability.

### 3.5. pH Conditions

pH significantly influences the interaction between proteins and polyphenols by altering the surface charge of both entities [4,15]. Thus, fluctuations in pH can modify protein conformation, influence polyphenol stability, and ultimately regulate binding affinity and interaction mechanisms. Proteins exhibit heightened sensitivity to pH variations, which can destabilize secondary and tertiary structures, consequently revealing concealed binding sites or modifying hydrophobic interactions [15,59]. β-lactoglobulin (β-LG) displays varying structural states based on environmental conditions: it exists as a dimer at neutral pH, a tetramer at pH 5.3, and a monomer at pH 2.5. Its affinity for polyphenols is directly impacted by these structural alterations [35].

The binding affinity and mechanisms of interaction vary markedly across pH ranges. Non-covalent interactions typically predominate in acidic environments [20]. At pH values below 7.0, proteins degrade more readily, exposing more binding sites and facilitating their interaction with phenolic chemicals [59]. Supporting this, Abdollahi et al. (2020) [69] observed that β-lactoglobulin displayed stronger affinity for ferulic acid when present in its monomeric form at pH 2.4. In contrast, protein conformations generally favor optimum binding at neutral pH. For instance, the soybean glycinin–EGCG and β-conglycinin–EGCG complexes exhibited their highest affinity at pH 7.0. In addition, polyphenols prevented heat-induced aggregation of lactoferrin in dairy systems, and the zein/ferulic acid complex exhibited its highest antioxidant activity at neutral pH, likely due to tyrosine exposure [4].

At alkaline conditions (pH > 7.0, particularly ≥9.0), polyphenols undergo oxidation to quinones and semiquinones, which subsequently form covalent bonds with protein side chains, resulting in stronger and irreversible complexes [13,20]. This covalent binding is reinforced by thiol group deprotonation, which enhances nucleophilicity [20]. Zein-ferulic acid complexes at pH 9.0 exhibited greater stability than those under acidic or neutral conditions, as Wang et al. (2022) [4] reported that ferulic acid had the most significant fluorescence quenching effect on zein at this alkaline level.

### 3.6. Temperature

Temperature significantly influences polyphenol–protein interactions, as thermal treatment effectively promotes or increases covalent bonding between these molecules [13]. This phenomenon is primarily ascribed to heat-induced denaturation of protein molecules, which reveals interior hydrophobic regions and may transition binding from predominantly non-covalent interactions to covalent conjugation [20,59]. Zhang et al. (2025) [13] indicated that heating enhanced covalent reactions between β-lactoglobulin and EGCG, resulting in stable monomeric and dimeric complexes at 90 °C, whereas Zhang et al. (2024) [37] found that higher temperatures made it easier for β-lactoglobulin and chlorogenic acid to covalently conjugate, stabilizing the resulting complexes. In addition to covalent interactions, temperature substantially affects non-covalent binding by altering its properties and strength. Disruption of hydrogen bonds facilitates the enhanced connectivity of certain phenolic compounds at elevated temperatures, hence augmenting hydrophobic interactions [20]. For example, as the temperature went up from 25 °C to 60 °C, the number of 5-O-caffeoylquinic acid molecules that were attached to bovine serum albumin went down, but the number of other phenolics that were attached to superoxide dismutase went up [20]. Moreover, in β-lactoglobulin–EGCG complexes, van der Waals forces and hydrogen bonding dominated above 60 °C, while electrostatic interactions became significant at temperatures exceeding 85 °C [61].

Temperature also controls the oxidation of polyphenols, in addition to binding. When the temperature rises, oxidation happens faster, which makes polyphenols more reactive and more prone to bind with proteins [61]. At the same time, temperatures above 60 °C can denature polyphenol oxidase, reducing enzymatic oxidation and altering the balance between enzymatic and non-enzymatic pathways [20]. However, excessively high temperatures may lead to polyphenol degradation or isomerization and can mask active hydroxyl groups on proteins, thereby diminishing the overall antioxidant activity [32].

The subsequent step is to examine the impact of these interactions on the secondary and tertiary structures of proteins, which in turn affects their overall stability and functionality, following the discussion of the primary factors that influence interaction.

## 4. Structural Modulation of Protein

### 4.1. Significance of Structural Changes

Protein structural changes induced by protein–polyphenol interactions are critically important because they directly determine the functional, nutritional, and biological performance of the resulting food matrices or delivery systems [3,4,12]. When polyphenols associate with proteins through non-covalent interactions or through irreversible covalent conjugation, they inevitably remodel the native protein conformation [3,4,12]. This structural remodeling provides the basis for many important results. Initially, it enhances functional attributes by increasing molecular flexibility. Proteins can more readily acclimate to diverse environments when transitioning from organized secondary structures (α-helices and β-sheets) to more disordered conformations (random coils) [42,43,70]. Increased flexibility facilitates the mixing of oil and water, hence enhancing emulsifying efficacy through the formation of thicker and more stable interfacial coatings [2,42]. Likewise, partial unfolding aids protein transport to air–water interfaces, thus improving foaming capability [35,42]. Depending on the interaction mode and concentration, these structural changes may also alter solubility, either by exposing hydrophilic groups or by promoting aggregation [12,42].

In addition, protein structural remodeling plays a central role in texture development and gel formation. In structured systems such as surimi and protein-based hydrogels, interactions with polyphenols, particularly tea-derived compounds, can regulate oxidation-induced cross-linking of myofibrillar proteins [62]. This guideline enhances the uniformity of gel networks, resulting in improved hardness and mechanical stability [3,71]. Conversely, insufficient structural control may lead to irregular aggregation and suboptimal gelling performance [3]. Structural modifications are also essential for stabilizing and delivering bioactive polyphenols, which are highly sensitive to light, heat, and digestive enzymes. Proteins can safeguard polyphenols by altering their conformation, thereby preserving their antioxidant properties under adverse conditions such as exposure to ultraviolet light [4,71,72]. Moreover, these modified protein structures can delay polyphenol release in the stomach and promote intestinal delivery, significantly enhancing bioaccessibility [12,71].

Changing protein structures changes food safety and health-related things. Polyphenol binding can change or hide IgE-binding epitopes. This might make proteins like egg white lysozyme and milk lactoferrin less likely to cause allergies [30]. Simultaneously, protein–polyphenol complexes frequently demonstrate synergistic bioactivity, exhibiting greater antioxidant capacity than either the protein or the polyphenol in isolation [2,43]. Finally, structural interactions between proteins and polyphenols are essential for preserving the physical stability of complex food systems. In emulsion-based beverages, alterations in surface charge distribution and hydrophobicity due to protein–flavonoid interactions enhance resistance to droplet coalescence and sedimentation during storage, thus improving product stability [2,42,44].

Overall, protein structural changes serve as both indicators and regulators of protein–polyphenol complex performance. To further clarify how these effects arise at the molecular level, the following sections examine alterations in protein secondary structure and tertiary structure.

### 4.2. Secondary Structure Changes

The secondary structure of a protein refers to the three-dimensional configuration of segments of the polypeptide chain. The components are mostly interconnected via hydrogen bonds between the amino and carbonyl groups in the backbone [5]. The structure comprises four principal components: α-helix, β-sheet, β-turn, and random coil [4,7]. Changes in these motifs caused by heat, oxidation, ultrasound, or contact with small compounds like polyphenols are not merely structural observations. Rather, they are critical determinants of how proteins interact with polyphenols, influencing the efficiency of complex formation, the stability of the resulting complexes, and functional properties such as solubility, emulsification, and antioxidant activity [5,29].

The α-helix is a rigid core that is kept together by hydrogen bonds between carbonyl oxygens and amino hydrogens. When proteins change, they usually get smaller [5,73]. This decrease is attributed to chain stretching and partial unfolding [42,43], mostly because polyphenol binding weakens hydrogen bonds. Such α-helix reduction exposes previously buried residues, providing reactive sites for polyphenol binding, thereby facilitating both covalent grafting and non-covalent interactions. This structural rearrangement directly affects the stability and functioning of the resulting complexes, increasing flexibility and potentially enhancing solubility and interfacial activity. For instance, the oxidation of myofibrillar protein (MP) from grass carp by hydroxyl radicals (·OH) significantly decreased α-helix content [3], whereas elevated concentrations of oxidized polyphenols (OC40 and OE40) diminished α-helix content by 17% and 20%, respectively, in a dose-dependent manner [73]. In a distinct instance, the non-covalent interaction of theaflavin (TF) with egg white lysozyme (LYZ) augmented α-helix content and diminished β-sheet content, resulting in a more compact conformation that could improve complex stability under particular functional conditions [30].

Changes in β-sheet structure are more variable, depending on the protein type, interacting compound, and binding mechanism [3,72]. When unfolding occurs, there is usually an increase because exposed hydrophobic residues encourage aggregation [3,6]. This aggregation can contribute to the formation of stable protein–polyphenol networks, enhancing gel strength, film formation, or particle stability. For instance, MP’s ·OH oxidation boosted its β-sheet and β-turn structures while decreasing its α-helix [3]. Similarly, EGCG binding to soy protein isolate (SPI) led to decreased α-helix and elevated β-sheet content [5]. In contrast, decreases in β-sheet are linked to more flexible, less ordered conformations, as observed in the interaction between goat lactoferrin (GLF) and pterostilbene (PTE), which lowered β-sheet while raising β-turn content [72]. In addition, succinylation of whey protein isolate (WPI) combined with soybean isoflavones (SI) gradually reduced β-sheet content [42]. These variations in β-sheet content reflect how secondary structure modulation governs accessibility of hydrophobic regions, influencing protein–polyphenol interactions and functional outcomes.

The random coil, representing the most flexible and disordered state, often increases when ordered structures are lost [7]. This shift, usually accompanied by higher β-turn content, enhances protein flexibility and facilitates multi-site polyphenol binding, thereby affecting complex formation efficiency and functionality such as emulsification, encapsulation capacity, and antioxidant potential [5,42,72]. In β-casein (β-CN) complexes with flavonoids (AG, LUT, QC, EGCG), random coil proportion rose significantly, indicating a looser conformation [43]. Likewise, ultrasound treatment of β-lactoglobulin (LG) drastically reduced β-sheet content and increased disordered structures. In the PPI-MD-EGCG ternary conjugate, the lowest α-helix and highest random coil levels were recorded, pointing to severe structural disruption [6]. These examples demonstrate that secondary structural modifications are not merely indicators of interaction; they also influence the design and efficacy of protein–polyphenol complexes.

To examine these modifications, spectroscopic methods, including Circular Dichroism (CD) and Fourier Transform Infrared (FTIR) spectroscopy, are extensively employed to delineate protein secondary structure changes resulting from polyphenol binding [70]. CD spectroscopy yields quantitative data regarding the relative proportions of α-helix and β-sheet structures, whereas FTIR analysis of amide bands facilitates the identification of conformational rearrangements among α-helix, β-sheet, β-turn, and random coil motifs [70,72,73]. X-ray diffraction (XRD) and Raman spectroscopy are two complementary methods that have also been used to show that there have been changes in the structure of protein–polyphenol groups [74]. All of these ways of looking at data together provide strong evidence of secondary structural regulation. This supports the idea that proteins and polyphenols interact with each other and affect how complexes are made, how stable they are, and how well they work.

### 4.3. Tertiary Structure Changes

The interaction between proteins and polyphenols induces significant alterations in the tertiary structure of proteins by modifying their three-dimensional folding and the microenvironments of their residues. Tertiary rearrangements represent the structural level where molecular binding events translate into complex stability and functional efficacy. Proteins typically undergo partial unfolding when they bind to polyphenols such as EGCG or resveratrol. This mechanism pulls on polypeptide chains, which brings aromatic residues like tryptophan, tyrosine, and phenylalanine to the surface. These residues were initially hidden in the hydrophobic core [43]. Because of this, molecules become more flexible, and interaction sites become easier to get to. As a result, protein–polyphenol complexes can stick to and rearrange themselves more quickly at oil–water or air–water interfaces. This makes them better at emulsifying and foaming [42].

At the same time, tertiary structural rearrangements cause a redistribution of surface hydrophobicity. This redistribution results from either the exposure of nonpolar groups during unfolding or their masking by hydrophilic polyphenol moieties [3]. The balance of hydrophobic exposure and masking is critical for influencing solubility, aggregation tendency, and colloidal stability. Excessive exposure to hydrophobic areas can result in intermolecular aggregation. In contrast, effective masking by polyphenol hydroxyl groups generally enhances dispersibility in aqueous environments and stabilizes the complexes [2]. Therefore, modulation of surface hydrophobicity provides an important structural basis for tuning stability–aggregation trade-offs in protein–polyphenol systems.

Polyphenol binding also alters the polarity of the chromophore microenvironment. These changes are commonly reflected by red-shifts or blue-shifts in intrinsic fluorescence spectra [2]. These spectrum shifts show changes in the shielding or exposure of residues within the tertiary fold and prove that stable ground-state complexes have formed. Importantly, changes in the microenvironment are intimately linked to oxidative stability. More open or remodeled conformations can more effectively accommodate and protect sensitive bioactive compounds from degradation [72]. Thus, fluorescence behavior serves as a useful indicator of the protective capacity of protein–polyphenol complexes.

In specific systems, polyphenols function as molecular connectors between protein chains. This interaction may result in structural compaction or the formation of larger aggregates through cross-linking, as indicated by changes in molecular size or density [30]. Such modifications influence both physical stability and biological processes. More compact or structurally constrained conformations may obscure or interfere with IgE-binding epitopes, thereby reducing immunological recognition and improving protein safety for sensitive consumers [30].

Tertiary structural modifications are generally detected by a combination of complementary analytical methods. Intrinsic fluorescence spectroscopy is frequently employed to observe alterations in the microenvironment of aromatic residues, as fluorescence quenching or shifts in wavelength indicate binding and tertiary structural rearrangements [42,70]. Surface hydrophobicity assays provide quantitative data regarding the exposure or concealment of non-polar regions on the protein surface [3,4]. UV-visible (UV-Vis) spectroscopy is also routinely used to detect changes in the absorbance behavior of aromatic residues, which provide additional evidence of microenvironmental polarity adjustments after polyphenol attachment [42,72]. Additionally, particle size investigations, such as dynamic light scattering, are used to assess the aggregation behavior and overall conformational compactness of protein-polyphenol complexes [12,72]. Collectively, these strategies contribute to a mechanistic knowledge of how tertiary structural modification influences complex formation, stability, and functional performance.

The secondary and tertiary structural changes in proteins in some studies are summarized in Table 1.

## 5. Technologies for Forming Protein-Polyphenol Complexes

**Figure 3:**
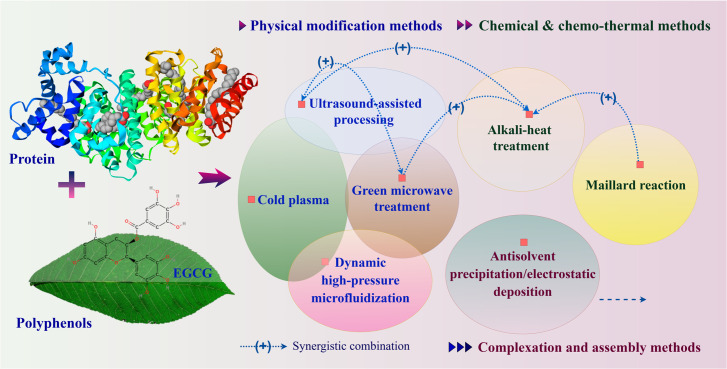
Processing technologies for protein-polyphenol interactions.

Based on the literature mentioned above, polyphenols may graft to proteins through non-covalent or covalent interactions. Indeed, covalent grafting forms permanent covalent connections, while non-covalent interactions mainly depend on ionic, hydrophobic, and hydrogen bonding mechanisms. To achieve this, there are various technological applications to promote protein-polyphenol interactions, including physical modification methods such as cold plasma technology, ultrasound, dynamic high-pressure microfluidization, and green microwave treatment. Additionally, chemical and chemo-thermal methods like alkali-heat treatment and the Maillard reaction, as well as other complexation and assembly methods such as antisolvent precipitation/electrostatic deposition, are employed and shown in Figure 3.

### 5.1. Cold Plasma Technology

In recent years, more attention has been directed towards cold plasma technology as a potential alternative to chemical and thermal treatment of food processing. This technology is particularly valuable for heat-sensitive components, such as bioactive compounds, without causing thermal degradation [75]. Cold plasma is a non-thermal method that is free of chemicals and water. It is environmentally friendly because it is generated from a complex mixture of high-energy free electrons, positive and negative ions, highly active free radicals, excited-state atoms and molecules, and ultraviolet photons [75,76]. From a theoretical standpoint, cold plasma can be visualized as a microscopic energy storm at ambient temperature wherein highly reactive oxygen (ROS) and nitrogen species (NOS) enhance the ionization of gases on the surface of the treated material under a high electric field [77]. Plasma-generated ROS can be attributed to modifying protein structures to form stable covalent bonds. Simultaneously, they are enhancing their phytochemical properties such as stability, antioxidant capacity, and bioactivity [24]. Under cold plasma treatment, the facilitated protein fibrillation mechanism stems from a decrease in the allergenicity of protein allergens [24,78]. The energy derived from cold plasma can disrupt weaker non-covalent bonds like hydrogen bonds and hydrophobic interactions. This disruption leads to partial unfolding of the protein structure. The treatment also causes partial unfolding of the protein structure and cleavage of the polypeptide chain of proteins, creating peptide fragments. These fragments are comparable to those produced by enzymatic hydrolysis. Post-treatment, the protein becomes activated and increases its reactivity to chemical reactions, especially with polyphenols. To demonstrate this mechanism, cold plasma has been successfully studied for ovalbumin and fine types of polyphenols in amyloid fibril formation. As confirmed by SDS-PAGE analysis, the presence of cross-linking between ovalbumin molecules by polyphenols was identified with ovalbumin dimers around 90 kDa and polymers over 140 kDa. Recently, Liu et al. (2025) [78] verified these findings. After 60 s of cold plasma treatment, gallic acid and EGCG exhibited higher covalent binding capacity with ovalbumin compared to other polyphenols. Further, the treatment of cold plasma made polyphenol grafting significantly reduce the IgE binding capacity of ovalbumin via an antigenicity test. As a result, after grafting, the functional properties of ovalbumin, such as antioxidant activity and emulsifying properties, were significantly enhanced [78].

### 5.2. Ultrasound Technology

High-intensity ultrasound technology was one of the most used methods to modify the molecular characteristics of food proteins, valued for its ability for high reproducibility, rapid processing times, enhanced rates of heat and mass transfer, and environmental sustainability [17,79]. The efficacy of ultrasound technology is also primarily driven by acoustic cavitation, which generates intense shear forces and micro-scale turbulence [80]. Cavitation bubbles form and then burst as soon as sound waves strike an object. This releases substantial energy that disrupts solvent molecules, generating abundant hydroxyl radicals (OH•), which in turn facilitate the oxidation of alkaline compounds [81]. At this time, mechanical shear, heating, dynamic agitation, intense shear forces, and micro-scale turbulence resulting from this process induce significant conformational changes in proteins, most notably unfolding proteins [82,83]. This structural alteration, in turn, exposes previously buried hydrophobic regions to the aqueous solvent, leading to a notable increase in the protein’s surface hydrophobicity [7,83]. Ultrasound treatment could both modify protein conformation simultaneously and disrupt existing protein structures, thereby enhancing protein-water interactions and improving functional properties [84]. However, this relationship is not always positive, for instance, by reducing extract entrapment efficiency or thermal stability, as the final effects are highly dependent on processing conditions [7,83]. Beyond merely altering the natural characteristics of proteins, these structural modifications also generate new opportunities for improving intermolecular interactions [82,85].

### 5.3. Dynamic High-Pressure Microfluidization

The application of dynamic high-pressure microfluidization (DHPM) to develop specific proteins and polyphenol complexes is becoming an important focus of research interest [86]. As an advancing homogenization technology, DHPM offers a combined processing mechanism that includes high-velocity impact, high-frequency vibration, cavitation, and shear. This process operates continuously while maintaining low processing temperatures and short processing times [86,87]. To modify food macromolecules, DHPM has been utilized in the preparation of nanoemulsions. According to Wang et al. (2023) [86], DHPM treatment at 120 MPa decreased the particle size of hemp seed protein (HSP)-gum Arabic (GA) bilayer emulsions from 869.03 nm to 260.02 nm, while also increasing zeta potential to −25.9 mV, which suggests improved colloidal stability. A similar reduced pattern was also observed in gliadin-rutin (G-R) complexes under 120 MPa treatment, where particle size reduced to 245.20 nm and a zeta potential increased to 21.76 mV. This indicates improved electrostatic repulsion [88]. It was noteworthy that these structural modifications were associated with enhanced interfacial protein adsorption. For example, HSP-GA emulsions exhibited a 92% increase in adsorbed protein content, rising from 19.44% to 37.35% after DHPM treatment [88]. In addition, in the case of emulsifying properties, the emulsifying activity index (EAI) and stability index (ESI) of G-R complexes increased from 35.93 m^2^/g to 69.46 m^2^/g and from 29.33 min to 43.59 min, respectively, under 120 MPa. The underlying rationale behind these observations is that DHPM induced conformational changes in the proteins. FTIR spectroscopy revealed shifts in the amide I (1660 cm^−1^) and amide II (1543 cm^−1^) bands, which confirmed new hydrogen bonding and hydrophobic interactions with polyphenols [87]. Rheologically, DHPM-treated emulsions exhibited a 2.5-fold and a 3-fold increase in viscosity and storage modulus (G’) (from 50 Pa to 150 Pa), respectively. This result also indicates the formation of stronger gel networks. Because of the possible effects of DHPM, it could be considered a unique method for forming protein-polyphenol interactions.

### 5.4. Green Microwave-Assisted Hydrolysis Technique

Unlike the previously mentioned non-thermal technology, the microwave-assisted hydrolysis technique is classified as a thermal process. In protein modification and complexation, microwaves are typically not used to directly facilitate the protein-polyphenol interaction itself. Instead, they act mainly as a thermal pretreatment step for protein extraction and modification. More specifically, microwaves are applied in the modification of the protein pre-treatment stage to hydrolyze difficult-to-process protein sources into hydrolysate. This process generates smaller and more soluble peptides with more reactive functional groups. This theory was in line with the findings of Shavandi and Ali [89], who reported that the application of microwaves hydrolyzes raw wool into wool hydrolysate. The yield of this process was approximately 90.0%. Under the same condition, Jafari et al. (2023) [90] also reported the formation of a silk wool and tannic acid complex as an in situ tissue adhesive through the utilization of polyphenol chemistry. In this study, the extraction of wool keratin was conducted using a microwave system. This process applied variable power levels up to 1800 W for a duration of 30 min at 180 °C with a ramp time of 30 min. Following this microwave-assisted hydrolysis treatment, the obtained keratin was successfully integrated with silk fibroin. The keratin was then cross-linked using tannic acid, a polyphenol, to form a tissue-adhesive hydrogel. Thus, the microwave-assisted hydrolysis technique plays an indirect role in effectively “unlocking” the potential of the protein for subsequent cross-linking reactions [89,90].

### 5.5. Alkali-Heat Treatment

Alkali-heat treatment is an effective method for synthesizing covalent protein-polyphenol complexes in emulsion delivery systems. This method works by exposing proteins to alkaline conditions and high temperatures [22]. This method offers a simple and effective way to make phenolic–protein complexes. When phenolic compounds are in an alkaline environment (like pH 9.0) and there is oxygen in the air, they oxidize to generate reactive semiquinones, which then turn into quinones. Electrophilic quinone intermediates can interact with nucleophilic amino acid residues on protein side chains. This interaction results in covalent cross-links, typically through C–S or C–N bonds [4]. In practice, under-treated by the alkali-heat processing method, the structure of ovalbumin (OVA) was induced in the formation of S-OVA, a more heat-stable form. In addition, this treatment leads to a transformation into a molten globule conformation, characterized by a looser and more flexible structure [84]. Additionally, the secondary structural analysis with the method of molecular docking and FTIR analyses demonstrated that hydrophobic interactions and hydrogen bonds act as the primary forces stabilizing these protein-polyphenol complexes [22]. The alkali-heat treatment also causes a reduction in β-sheets and an increase in irregular curling and β-turns [91]. Based on these molecular interactions, by encasing polyphenols in an amorphous state within its hydrophobic core, the treated protein mechanistically operates as an effective carrier, thereby enhancing their solubility and bioavailability [91]. This structural organization facilitates the formation of complexes, especially covalent ones, which markedly increase the stability and bioaccessibility of polyphenols by protecting them from degradation during digestion [12]. Consequently, the conjugation of polyphenols enhances the antioxidant capacity of the complex; however, this enhancement may be partially constrained by hydrogen bonding, which can diminish the number of available reaction sites. Extending beyond antioxidant properties, the complexes exhibit improved techno-functional characteristics. They demonstrate enhanced emulsifying activity and emulsion stability, which are critical for food applications [19].

To create an optimal setting for covalent conjugation, the operational parameters of alkali-heat treatment are tailored to develop specific functional outcomes and to vary markedly among various protein-polyphenol complexes [92]. Regarding the effect of this treatment, a well-documented process is the transformation of ovalbumin, pea protein isolate, and soy protein isolate into their more heat-stable form [24,84,93]. This transformation took place at a targeted pH of 9.0 and a temperature of 55 °C for 24 h. However, in contrast to this general trend of applying explicit heat, several studies utilize alkaline conditions at ambient temperatures to achieve conjugation. For instance, the gelation of duck egg white was induced by 0.57% (*w*/*w*) NaOH, resulting in a pH above 12.0, followed by storage at 25 °C for three days to reach equilibrium [22]. A comparable approach was noted in research on the interaction between zein and ferulic acid [4], soy protein isolate with catechin [12], and coconut protein isolate with ferulic acid [1]. In this study, interactions were facilitated at a pH of 9.0 by stirring for 12 to 24 h at room temperature. Therefore, the proper choice of specific parameters of alkali-heat treatment is crucial for effective conjugation and for optimizing the desired structural as well as functional modifications in the complex.

### 5.6. Maillard Reaction 

The Maillard reaction between carbonyl groups of sugars and amine groups of amino acids leads to desirable modifications in food properties [94]. It is a safe and effective method used to modify proteins to improve their physicochemical and functional properties [25]. Indeed, the combination of Maillard and polyphenol covalent reactions may produce synergistic effects on the structural properties of proteins [6]. These modifications typically initiate at the secondary structure level, resulting in a reduction in α-helices and an increase in unordered structures, such as random coils [95,96,97]. Thus, researchers have attempted to investigate the effects of different protein types on protein-polyphenol complexes during the Maillard reaction and their potential applications [97,98,99]. Under the Maillard reaction, Liu and colleagues [95] demonstrated a sophisticated multi-step process that involved the further modification of a preformed lactoferrin-chlorogenic acid conjugate via the Maillard reaction. Their findings indicated a direct synergy that occurs whereby the Maillard reaction enhances the properties of a pre-functionalized protein-polyphenol system. The study also revealed that glycosylation through the Maillard reaction synergistically boosted the conjugate’s antioxidant potential and thermal stability. Additionally, these results highlighted that the covalent linkages formed during the Maillard reaction are essential for developing a robust protective matrix [100]. This outcome above is in line with the recent study of Wang et al. (2025) [94]. Their research highlighted the synergy in Maillard reaction products, which were formed between different proteins and xylo-oligosaccharides for encapsulating flavonoids from sea buckthorn pomace. The study proved that the Maillard reaction creates a delivery system that can more than double the potential bioavailability of the encapsulated polyphenols [94]. As compared to that of the free extract, microencapsulation using Maillard reaction products enhanced the bioaccessibility and protective effect of flavonoids during simulated gastric digestion [94]. Collectively, the Maillard reaction is a powerful and versatile platform technology for synthesizing high-performance protein-polyphenol complexes.

### 5.7. Antisolvent Precipitation/Electrostatic Deposition Method 

Beyond covalent conjugation, another significant process technology for the formation of protein-polyphenol systems is antisolvent precipitation followed by electrostatic deposition [23]. This dual-phase methodology fabricates multi-component, core–shell biopolymer nanoparticles designed for encapsulation and delivery. This technique demonstrates optimal efficacy for hydrophobic proteins like zein, which is soluble in alcohol-water solutions but insoluble in water [101]. In the initial step, a hydrophobic protein (core) and polyphenol, such as hesperetin [23] or curcumin [102], are co-dissolved in an ethanol solution and then mixed rapidly with an aqueous phase. The induced change in solvent polarity facilitates the co-precipitation of both components, leading to the formation of a protein core that physically encapsulates the polyphenol. As can be observed by spectroscopic analyses, this entrapment is mainly stabilized by hydrogen bonding and hydrophobic interactions [103]. Subsequently, in the latter deposition step, a charged polysaccharide is introduced to the nanoparticle dispersion. Through electrostatic deposition, the anionic polysaccharide forms a protective hydrophilic shell around the cationic zein core (at pH < pI). As a result, the core–shell greatly enhances the colloidal stability of the nanoparticles over a wide range of pH, ionic strength, and thermal conditions, while also enhancing the bioaccessibility and antioxidant activity of the encapsulated polyphenol [102].

## 6. Synergistic Bioactivities of Protein-Polyphenol Complexes

**Figure 4:**
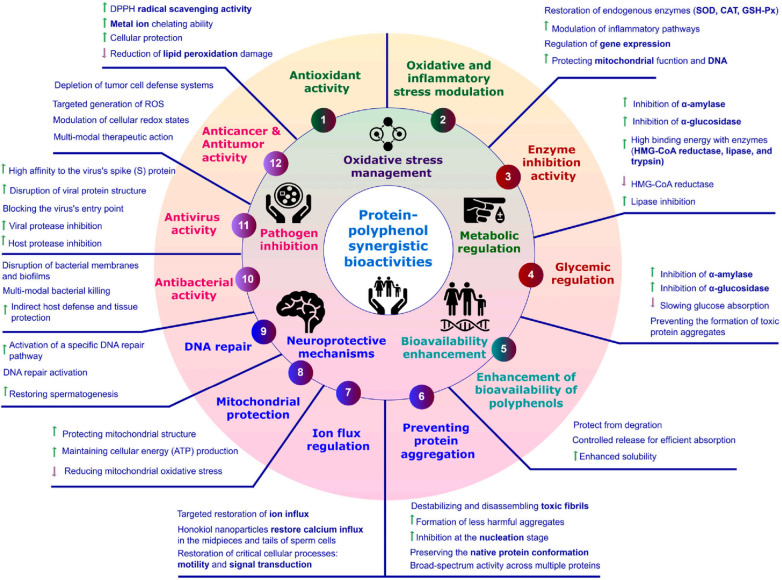
Mechanistic diagram of biological activities in polyphenol-protein complexes. ↑ and ↓ indicate an increase and decrease in the corresponding biological activities, respectively.

Beyond their conventional role in stabilizing bioactive compounds, protein–polyphenol complexes have recently attracted attention as multifunctional platforms with diverse biological activities (Figure 4). These complexes often show synergistic effects that enhance antioxidant capacity, anti-inflammatory properties, metabolic regulation, pathogen inhibition, and bioavailability. Here, we review key findings on the synergistic bioactivities, quantitative results, and mechanisms underlying these effects (Table 2).

### 6.1. Antioxidant and Anti-Inflammatory Properties

The oxidative degradation of unsaturated fatty acids negatively impacts food quality and human health. Therefore, developing effective antioxidant systems is necessary when dealing with the solubility limitations of free polyphenols and the low activity of native proteins [107]. In this context, protein-polyphenol complexation addresses these issues by transforming both components into synergistic functional ingredients [108]. As shown in Table 2, this interaction leads to a significant increase in antioxidant capacity, for instance, when complexed with apple polyphenols, the radical scavenging activity of soy protein isolate increased from 4.64% to 89.48% [29]. More importantly, this enhancement extends well beyond in vitro chemistry to cellular environments. In these biological systems, the protein carrier performs dual functions: it effectively stabilizes polyphenols while restoring endogenous enzymes (SOD, CAT, GSH-Px). Additionally, it significantly reduces lipid peroxidation, such as MDA levels [29]. Based on these analyses, the polyphenol-protein complexes effectively restored the activity of endogenous antioxidant enzymes, especially with respect to the WPI-AP complex which increased Glutathione Peroxidase (GSH-Px) levels to 122.31 U/mg. Mechanistically, this extended activity is explained by the protein matrix’s “structural shielding” effect. The key is covalent cross-linking, which acts like a protective barrier around unstable polyphenols like catechin from thermal and environmental degradation during storage [12]. The key is covalent cross-linking, which acts like a protective barrier around unstable polyphenols like catechin from thermal and environmental degradation during storage [12]. However, one important consideration in the complexes is that this structural stabilization presents a functional trade-off known as the masking effect. More generally, complexation enhances stability but comes with a trade-off. The protein matrix masks the reactive hydroxyl groups present in polyphenols. In turn, this reduces direct radical scavenging compared to free forms (e.g., 74.21% for the SPI-EGCG complex vs. 87.04% for free EGCG) [9]. Taken together, the enhanced value of these complexes has drawn a lot of benefits, including their immediate reactivity and their ability to preserve and provide antioxidant potential under physiological and processing conditions.

### 6.2. Metabolic Regulation via Enzyme Inhibition

Regulating glucose absorption by inhibiting carbohydrate-hydrolyzing enzymes is a critical strategy for the management of type 2 diabetes [104]. Native polyphenols inhibit these enzymes; however, their efficacy is often limited by poor solubility and stability. Such issues can be addressed by using protein complexation to alter the structure of the inhibitor [105]. As shown in Table 2, the complexation of curcumin nanoparticles with zein through antisolvent precipitation (Cur-ASP) significantly enhanced their inhibitory potential against key digestive enzymes (α-amylase and α-glucosidase). This complex exhibited the highest inhibition rates at 100 mg/mL, with 68.67% for α-amylase and 58.30% for α-glucosidase, which are similar to the standard drug acarbose. Notably, when tested against β-glucosidase, the complex proved more effective than native curcumin [105]. This synergistic enhancement stems from the protein-mediated synthesis of nanoparticles, which makes curcumin more soluble and increases its specific surface area. In this way, the polyphenols in the complex can more easily reach and bind to the enzyme’s active site, leading to stronger inhibition of digestive enzymes and enhanced antioxidant activity [29,105]. In this line, Dai et al. (2023) [12] showed synergistic enhancement of the complex during digestion, where the protein in the complex is hydrolyzed into antioxidant peptides. These peptides work alongside the polyphenols to enhance cellular production and regulate metabolic signaling pathways.

### 6.3. Antimicrobial Efficacy and Pathogen Inhibition

Polyphenols, such as epigallocatechin gallate (EGCG) [25], are natural compounds with strong antibacterial activity and their interactions with proteins are crucial. They can damage bacteria directly because they induce the production of reactive oxygen species (ROS) or deplete bacterial antioxidant defenses [25]. Additionally, polyphenols can interact with metals to form hybrid nanostructures with proteins, which enhances their bactericidal activity [109]. They also strengthen host defenses by regulating proteins such as claudin-2 to suppress inflammation and maintain tissue integrity [110]. Chen et al. (2024) [25] reported that the self-assembling Cu–EGCG nanocomposite uses EGCG as a chelating agent for Cu^2+^ ions, forming a metal–polyphenol network that interacts with proteins. These combined mechanisms disrupt bacterial membranes, inhibit biofilm formation, and enhance antibacterial activity. In vitro, Cu–EGCG (400 μg/mL) demonstrated killing efficiency exceeding 90% against six strains (*E. coli*, *S. aureus*, MRSA, E.C. EIEC, *P. aeruginosa*, and *Salmonella*). Combined with NIR, this system achieved over 99% killing efficiency against MRSA. What is more, morphological analysis by SEM revealed that single components caused only slight surface depressions. In contrast, the Cu-EGCG complex caused significant surface collapse. When combined with NIR, this effect intensified, destroying bacterial structures and causing intracellular protein leakage.

In addition, in vivo MRSA-infected wound models showed accelerated healing, minimal bacterial retention, and reduced inflammation, with preliminary safety assessments suggesting low toxicity. Other studies further highlight the synergy of polyphenol–protein interactions. Wang et al.’s case study from 2022 [111] also shows that gallic acid (GA) and resveratrol (Res) hydrogels can be made in just 5 min through hydrogen bonding and π–π interactions, creating protein-interactive polyphenol assemblies. These hydrogels release GA and Res, reduce ROS and nitric oxide (NO), and regulate inflammatory cytokines. The formulations showed strong antibacterial activity (MIC 3 mg/mL for *E. coli*, 4 mg/mL for *S. aureus*) and demonstrated in vivo wound-healing efficacy. Collectively, these studies show that polyphenol–protein interactions generate multimodal and synergistic antibacterial effects, directly eradicating bacteria while protecting host tissues. Advanced formulations such as Cu–EGCG nanocomposites and GA/Res hydrogels highlight that combining polyphenols with proteins significantly improves antibacterial efficacy and therapeutic potential.

### 6.4. Bioavailability Enhancement

From a nutritional perspective, bioavailability is the proportion of a substance in a given food that the body can effectively utilize and is therefore a matter of nutritional efficacy [112]. In the body, bioavailability consists of multiple processes, including liberation from a food matrix, absorption, distribution, metabolism, and elimination through different mechanisms as well [58]. As is the case with protein-polyphenol complexes, their formation enhances the polyphenol bioavailability through three specific mechanisms. First, the complexes serve to protect polyphenols from degradation by environmental factors such as temperature, harsh pH levels, and digestive enzymes within the body [13]. Second, these complexes present with the following, which contribute to inhibiting the uncontrolled release of polyphenols in the stomach. This procedure helps to slow and regulate polyphenol release in the intestine, where absorption is more efficient [113]. Third, the presence of protein binding enhances the solubility of polyphenols, which typically exhibit low water solubility, particularly within the micellar environment of the small intestine, resulting in improved absorption of protein [42].

The polyphenol bioavailability was confirmed by the recent study of Dai et al. (2023) [12] with respect to non-covalent and covalent complexes of soy protein isolate and catechin. Through an in vitro digestion model test, covalent complexes of isolated soy protein and catechin demonstrated a significant increase in catechin bioavailability up to approximately 86%, compared to free catechin, which was about 30%. This occurs due to catechin being strongly attached to the protein through irreversible covalent bonds. As a result, this conjugation helps inhibit interaction with degrading enzymes and facilitates slow release [12]. Similarly, the combination of apple polyphenol (AP) complexes and soy protein isolate (SPI), chickpea protein (CP), tartary buckwheat protein (TBP), and whey protein isolate (WPI) was evaluated to improve the bioavailability of AP. Specifically, the complex of AP with TBP showed the highest polyphenol bioavailability at 74.01%, followed by WPI-AP (54.25%), CP-AP (47.57%), and SPI-AP (43.59%), whereas free AP was 40.83% [29]. In the latter case of curcumin, a kind of hydrophobic bioactive component, the application of which is limited due to its instability and low bioavailability. Therefore, in order to alleviate this problem, a new pH-controlled emulsion was introduced. This system was proven to be an effective, pH-controlled carrier for curcumin. It showed the highest curcumin loading efficiency (92.30%) and improved curcumin bioavailability, with slow release in simulated intestinal fluid [6].

Recently, Ji et al. (2024) [71] well-illustrated this concept of bioavailability through the co-delivery of curcumin and EGCG within soy protein fibrils. In this study, soy protein fibrils acted as the primary structural component, promoting effective internalization and enhancing deep intracellular transport of curcumin and EGCG into Caco-2 colorectal cancer cells [71]. Indeed, the hydrogel framework provided a protective microenvironment, effectively protecting Cur and EGCG from external stressors, such as prolonged thermal exposure. After thermal treatment for 120 min, the retention rates of curcumin and EGCG reached 95.27% and 70.27%, respectively, compared to 53.13% and 13.32% for their free forms. Moreover, driven by this superior stability and delivery, the complex demonstrated potent anticancer activity. At a curcumin concentration of 5 µg/mL, the viability of colorectal cancer cells decreased to 8.29%, whereas free polyphenols maintained cell viability above 95%. Notably, the use of soy protein fibrils achieved remarkable encapsulation efficiencies, reaching 97.71% for Cur and 91.02% for EGCG. As a result, a maximal fraction of the active compounds was accessible at the target site. Taken together, the above results also highlighted that protein-polyphenol complexes could be used as a new kind of encapsulation material and provide new ideas for the enhancement of bioavailability and delivery of bioactive components.

## 7. Applications in Food Systems and Industrial Challenges

### 7.1. Active Packaging Films

**Figure 5:**
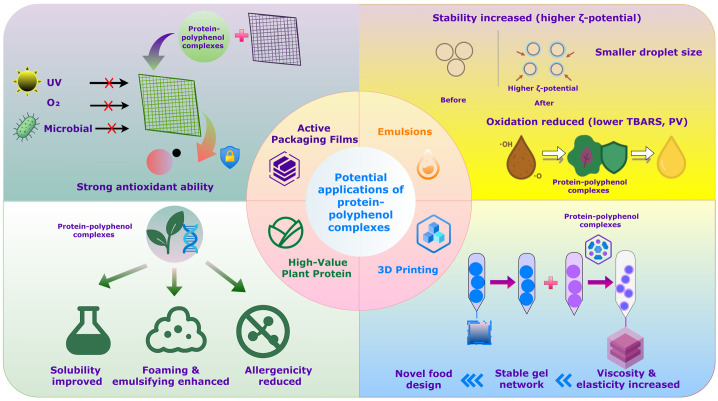
Potential applications of protein-polyphenol complexes in the food systems.

Protein–polyphenol complexes have been effectively incorporated into edible films. That offers a sustainable alternative to synthetic plastics while providing active preservation functions for packaged foods, as illustrated in Figure 5 [33,74]. These films not only enhance barrier properties against UV–visible light, oxygen, and water vapor but also improve mechanical strength and deliver direct antioxidant and antimicrobial effects at the food surface [33,74]. For instance, in a study by Zhou et al. (2021) [74], incorporating mangosteen peel extract (MPE) into soy protein isolate (SPI) films doubled the total phenolic content from 1.5 to 3.0 mg GAE/g at 15.0% MPE, significantly increased opacity (*p* < 0.05) to block UV–Vis light, and improved tensile strength and elongation at break through hydrogen bonding and hydrophobic interactions between polyphenols and proteins. Similarly, according to Feng et al. (2025) [33], ternary complexes of luteolin, egg-white protein, and rice glutelin embedded in chitosan films reduced UV transmittance, ranging from 2.75% to 22.10%, with unfolded egg-white proteins showing the strongest UVA-blocking effect. As well, these conjugate-composite films reduced water vapor permeability, achieving values of 2.321 ± 0.054 g⋅mm/(m^2^⋅h⋅kPa). Preservation studies further confirmed their functional benefits. In cherry tomatoes, which are stored for 10 days, films such as H5C retained 23.65 ± 0.70 mg/100 g vitamin C, maintained stable pH and soluble solids. Moreover, their surface appearances were preserved better than air-exposed controls [33]. Conjugate-based films also prevented rice cake surface cracking within 24 h and maintained pH and titratable acidity at levels comparable to polyethylene films [33]. Overall, these findings demonstrate that protein–polyphenol complexes serve as multifunctional active packaging with strong antioxidant and antimicrobial effects, thereby improving food quality and extending shelf life.

### 7.2. Food Emulsion Stabilization

Emulsions are ubiquitous in the food industry. Emulsions serve as delivery systems for lipophilic bioactive compounds, modifying texture and improving product stability [1,114]. Proteins are widely used as emulsifiers because of their amphiphilic nature. However, they are often sensitive to environmental stressors like pH, ionic strength, and temperature, which limits their application [1,2,29]. Protein-polyphenol interactions significantly contribute to the formation and stabilization of these complex systems. Polyphenol–protein complexes play a crucial role in improving the stability of oil-in-water (O/W) emulsions, which are essential for preserving food quality and extending shelf life, as shown in Figure 5 [21,105,106,107,108]. These complexes improve stability by augmenting electrostatic repulsion and steric hindrance among emulsion droplets, creating denser interfacial layers at the oil-water interface, and functioning as radical scavengers and metal ion chelators [114,115,116]. A range of studies has demonstrated both physical and oxidative stabilization effects, supported by quantitative data. For example, the incorporation of resveratrol into whey protein isolate increased the absolute ζ-potential and decreased droplet size, thereby improving emulsion stability [2]. After 10 days of storage, the carbonyl content of unadsorbed proteins (UAP) and adsorbed proteins (AP) decreased significantly at 2.0 mM resveratrol (UAP: 4.33 µmol/mg; AP: 5.13 µmol/mg) compared to the control (UAP: 6.68 µmol/mg; AP: 8.21 µmol/mg) [2]. In addition, N’-formyl-L-kynurenine (NFK) levels in AP declined by approximately 13%, 19%, 22%, and 27% as resveratrol concentrations increased (0.2, 0.4, 0.8, and 2.0 mM, respectively). These reductions confirm that resveratrol molecules, concentrated at the oil-water interface through binding to AP, function as principal antioxidant sites. This conclusion is similar to the result that tannic acid (TA) with hydrolyzed soy protein isolate (HSPI) improved emulsifying and oxidative stability. The HSPI/TA complexes exhibited reduced peroxide value (PV) and thiobarbituric acid-reactive substances (TBARS) compared to HSPI or soy protein isolate (SPI). The stability ranking was as follows: HSPI/TA0.06 > sodium caseinate (SC) > HSPI > SPI. This enhancement stemmed from augmented surface charge and the establishment of a particle-stabilized interfacial membrane, which prevented pro-oxidants from reaching oil droplets [116]. The combination of epigallocatechin-3-gallate (EGCG) with β-lactoglobulin nanoparticles (β-lgNPs) also provided significant advantages. Lutein emulsions stabilized with β-lg-EGCG nanoparticles demonstrated an 87.2% retention of lutein after 30 days of storage, suggesting enhanced chemical and physical protection relative to alternative formulations [18].

Stabilization occurred through the development of a dense interfacial layer, thereby reducing droplet aggregation by steric repulsion [33]. The stabilization mechanism involved the creation of a thick interfacial layer that reduced droplet aggregation through steric repulsion. In a separate study, ferulic acid (FA) was covalently grafted onto coconut protein isolate (CPI) via alkaline treatment, resulting in its rapid migration to the oil–water interface, where it established a thick and elastic layer [1]. This modification enhanced antioxidant activity by increasing DPPH and ABTS radical scavenging and improved centrifugal stability, effectively inhibiting lipid oxidation during a 15-day storage period. Polyphenol–protein complexes exert their effects across a wide range of protein sources. Apple polyphenols (AP) combined with soy protein isolate (SPI), whey protein isolate (WPI), chickpea protein (CP), and Tartary buckwheat protein (TBP) enhanced the antioxidant capacity of the proteins, protected HepG2 cells against H_2_O_2_-induced oxidative stress, and restored antioxidant enzyme activity. The protection ranking was WPI-AP > TBP-AP > SPI-AP > CP-AP, while bioaccessibility improvement followed the order TBP-AP > WPI-AP > CP-AP > SPI-AP [29]. This study supports the notion that proteins function as carriers that prolong the gastrointestinal release of polyphenols. Quercetin (QC) combined with potato protein (PoP) resulted in hybrid nanoparticles that effectively stabilized Pickering emulsions for extended periods, lasting several months [44]. Increasing QC content resulted in a reduction in ζ-potential and an increase in droplet size, indicating a stronger electrostatic stabilization correlated with polyphenol concentration [44]. The findings indicate that polyphenol–protein interactions provide an effective approach to enhance emulsion stability via integrated physical and antioxidant mechanisms. The incorporation of polyphenols alongside various protein sources offers opportunities for the development of stable, functional food emulsions with prolonged shelf life.

### 7.3. High-Value Plant Protein Formulation

The global demand for plant-based proteins is rapidly increasing because consumers seek sustainable and healthy alternatives to animal proteins [29,116]. However, many plant proteins, such as soy, chickpea, and wheat germ proteins, show poor solubility and weak functional performance, which restricts their use in food systems [5,116]. Interactions with polyphenols, particularly non-covalent binding, represent a green, simple, and effective strategy to improve these limitations and to transform plant proteins into high-value functional ingredients comparable to their animal-derived counterparts [116].

The functional enhancement of plant proteins arises from molecular-level structural modifications induced by polyphenol binding. First, polyphenols disrupt compact protein conformations through hydrogen bonding, hydrophobic interactions, and electrostatic forces [29,117]. These unfolding decreases ordered secondary structures like α-helices and β-sheets, while increasing disordered structures like β-turns and random coils for proteins such as SPI [114], wheat germ protein (WGP) [21], and chickpea protein isolate (CPI) [117] upon interaction with polyphenols, which confer greater flexibility. Second, polyphenols can also alter the protein’s surface characteristics. This effect can be ascribed to masking hydrophobic patches or introducing hydrophilic hydroxyl groups, thereby reducing surface hydrophobicity [116,117]. At the same time, they increase the net negative surface charge, which strengthens electrostatic repulsion between molecules and reduces protein aggregation [21,116]. Together, these effects markedly enhance protein solubility [21,43]. Third, the improved solubility and conformational flexibility allow proteins to adsorb and rearrange more effectively at air–water and oil–water interfaces, which directly improves emulsifying and foaming capacity as well as stability [21,117].

Application studies confirm these mechanisms. In soy protein isolate (SPI), interactions with epigallocatechin gallate (EGCG) or tannic acid (TA) unfolded the protein and enhanced emulsifying activity and stability indices, enabling SPI to perform comparably to sodium caseinate [114,116]. In chickpea protein isolate (CPI), treatment with the flavonoid naringin doubled foaming capacity (from 42.6% to 88.0%) and strengthened foam stability. This improvement is linked to reduced α-helix content and altered surface charge so that it facilitates the formation of a more robust interfacial film [117]. In accordance with Liu et al.’s (2024) result [21], wheat germ protein (WGP) complexed with chlorogenic acid or EGCG displayed higher solubility and emulsifying ability because of their increased structural flexibility and reduced hydrophobicity. In a comparative study, tartary buckwheat protein (TBP) had the highest binding capacity with apple polyphenols (AP) among various plant and animal proteins [29]. The TBP-AP complex protected polyphenols during digestion, giving a bioaccessibility of 74.01%, nearly double that of free apple polyphenols (40.83%). These findings suggest that polyphenol modification is a powerful method for upgrading the functional value of plant proteins, enabling their use as effective foaming and emulsifying agents and as substitutes for animal proteins in diverse applications, as illustrated in Figure 5. The outcome, however, is that both the protein source and the polyphenol type play critical roles, as their unique features determine the extent and mechanism of functional enhancement.

### 7.4. 3D Food Printing Performance

According to Zhang et al. (2025) [118], food 3D printing is a transformative technology because it enables the production of foods with customized shapes, textures, and nutritional profiles. This technology can improve unconventional protein sources’ visual and textural properties, such as insect proteins. Therefore, it can increase consumer acceptance. However, many protein-based inks face significant limitations because of their inadequate rheological characteristics. Furthermore, they lack the extrusion fluidity and structural stability needed to maintain shape after printing [119].

The incorporation of polyphenols can significantly alter the characteristics of protein gels and make them suitable for 3D printing, as shown in Figure 5. The principal mechanism is the reinforcement of the gel network, and polyphenols like EGCG promote the aggregation of protein molecules within the gel matrix. As cross-linkers, polyphenols facilitate the development of a denser, layered, and porous network. As a result, this establishes a strong internal framework and significantly improves structural stability and resistance to gravitational deformation.

These modifications directly influence rheological properties. For example, the addition of EGCG to honey bee pupa protein (HBPP) gel increased viscosity, storage modulus (G′), and loss modulus (G″). Therefore, that shifted the gel toward a more solid-like behavior essential for self-support after printing. Importantly, the gel maintained shear-thinning (pseudoplastic) behavior. That helped to allow smooth and consistent extrusion through the printer nozzles without requiring excessive pressure.

At the molecular level, polyphenols induce conformational changes in protein and promote the transition from ordered secondary structures (α-helix, β-sheet) to more disordered forms (random coil, β-turn). This unfolding is stabilized by hydrogen bonding and hydrophobic interactions and allows protein chains to rearrange so that they establish a stronger and more stable network architecture. Specific studies on HBPP gel illustrate this effect: pure HBPP gel lacked structural integrity, and printed hollow cylinders collapsed immediately. Adding ≥ 2% EGCG significantly improved self-supporting ability and enabled stable hollow cylinders to form that did not collapse. Rheological and textural analyses showed that viscosity, G′, G″, Young’s modulus, and resistance to creep deformation all increased, but extrusion performance remained unaffected. SEM images further verified that EGCG induced protein aggregation and transformed the compact microstructure into a denser, layered, and porous network. That helped provide necessary structural support.

Beyond HBPP, other protein-polyphenol systems have been studied for 3D food printing. Casein-hyaluronic acid emulsion gels were developed with high water-holding capacity and excellent rheological properties, and high internal phase emulsions crosslinked non-covalently with soy protein isolate and tannic acid were also prepared for printing applications [119]. In addition, the overall printability of food pastes depends strongly on their rheological behavior, and both the gel’s spatial structure and its water state play a decisive role, as shown in studies using honeybee pupa protein and soybean dietary fiber gels [120]. Furthermore, the composition and physical properties, particularly the protein and chitin content, influence the printability of insect-derived gels [121]. The synergistic combination of κ-carrageenan and L-lysine has also enhanced the printability of peach gels and pointed out the value of material elasticity in the printing process [122].

These findings show that polyphenol-mediated protein modification is an effective way to design food inks for 3D printing. By controlling protein aggregation, it creates gels with the right balance of strength, elasticity, and fluidity, enabling the use of diverse protein sources, including sustainable options like insect proteins.

### 7.5. Commercialization and Industrial Challenges

At present, most studies on the application of polyphenol–protein complexes are still conducted at the laboratory scale. The industrialization of protein–polyphenol complexes remains constrained by persistent challenges related to raw material quality, processing scalability, product stability, and consumer acceptance. Polyphenols often exhibit low water solubility [42] and high sensitivity to environmental stresses (UV irradiation, enzymatic activity, etc.), while many natural proteins show rigid globular structures [116]. Protein isolation and purification further impose technical and economic burdens [123]. In addition, consumer neophobia limits the adoption of proteins from novel sources (such as honey bee pupa protein) [118]. During processing, traditional methods used for preparing protein–polyphenol complexes frequently suffer from low efficiency and long reaction times, which restricts large-scale commercialization. While utilizing ultrasound can enhance efficiency in alkali or free radical treatments, it can decrease grafting efficiency in enzyme-mediated conjugation by lowering the enzyme’s activity [121]. In food applications, undesirable color changes [121,122], insufficient lipid oxidative stability and excessive protein cross-linking at high polyphenol concentrations compromise emulsifying performance and product quality [124]. Moreover, current evaluations rely largely on in vitro antioxidant assays, which do not adequately explain the in vivo behavior of protein–polyphenol complexes. Future research must therefore integrate scalable green processing strategies and physiological validation to support the development of stable, functional, and commercially viable protein–polyphenol complex-based products.

## 8. Limitations in Interaction Mechanisms and Technological Applications

Unfortunately, despite numerous studies that have been developed in the past five years, the precise mechanisms of how proteins interact with polyphenols are not fully understood, particularly regarding covalent bonding under natural conditions. Factors influencing binding efficacy, such as the structure of polyphenols (e.g., number of -OH groups, molecular weight) and the identification of specific binding sites on proteins, need to be further examined. Another major gap in current studies is the absence of a comprehensive analysis of these complexes, including identifying the specific products and by-products (such as protein-polyphenol adducts formed during autoxidation), necessitating advanced and sensitive analytical techniques. It is also important to emphasize that traditional analysis methods need long reaction times and the use of chemical reagents. Creating stable protein-polyphenol complexes calls for efficient and environmentally friendly methods.

Notwithstanding the biological activities results of complexes, this is still complicated in the autoxidation of polyphenol, which produces reactive intermediates such as hydrogen peroxide (H_2_O_2_) and *o*-quinones. These intermediates can lead to protein oxidation and the formation of undesirable products. In addition, although complexation can protect polyphenols, the binding process itself can sometimes reduce their bioactivity. The formation of extensive hydrogen bonds between polyphenols and proteins may decrease the number of effective antioxidant reaction sites. These interactions can negatively affect the digestibility of the complexes and may impart undesirable astringency to food products. By overcoming these barriers, opportunities and future research could unlock the full potential of protein-polyphenol complexes.

## 9. Opportunities and Future Perspectives

### 9.1. Opportunities

The greatest opportunity in polyphenol–protein complexes is using proteins as delivery vehicles to protect polyphenols from degradation during processing, storage, and digestion, which enhances their bioavailability. Covalent complexes can enhance catechin bioaccessibility by approximately 86%. These complexes can act as controlled-release systems and slowly release polyphenols in the intestine for more effective absorption. Serum albumins, for example, human serum albumin and bovine serum albumin, are becoming versatile carriers for targeted drug delivery. Further, forming complexes with polyphenols can improve protein functional properties, including emulsifying, foaming, and solubility. For example, a pea protein isolate–maltodextrin–EGCG ternary conjugate showed the highest emulsifying ability and antioxidant activity. Especially, polyphenols may mask or alter epitopes on the protein surface, which reduces IgE/IgG antibody binding and mitigates allergic responses. Covalent conjugation with polyphenols can reduce the allergenicity of proteins such as ovalbumin and β-lactoglobulin. Thus, such modified proteins may serve as natural, safe, and effective emulsifiers or foaming agents in the food industry.

To date, emerging processing technologies such as ultrasound, high hydrostatic pressure, and cold plasma provide efficient approaches to create polyphenol–protein complexes with improved performance. Ultrasound treatment increased the binding affinity between whey protein isolate and EGCG by more than tenfold, while cold plasma enabled covalent conjugation within 60 s without chemical reagents. It is worth mentioning that polyphenol–protein complexes can also be applied as functional ingredients in plant-based protein beverages and nutrient delivery systems. They combine health benefits such as antioxidants, anti-inflammatories, and anticancer effects. In particular, the soy protein isolate–catechin covalent complex exhibited stronger antioxidant activity than its non-covalent counterpart. Therefore, this demonstrates its potential for functional food applications.

### 9.2. Future Perspectives

Although in vitro studies have demonstrated encouraging outcomes, future research must validate these findings in in vivo models. This involves evaluating the efficacy of the complexes in mitigating allergenicity, improving bioavailability, and comprehending their effects on gut microbiota, but does not explain in vivo biological effects adequately. Future research must clarify the absorption, distribution, and metabolism of protein-polyphenol complexes through animal and human studies, and it must verify their physiological relevance beyond the cellular level. Simulated digestion models also differ from real human digestion and require further validation. Further studies are needed to elucidate the molecular mechanisms of polyphenol–protein interactions. They should use advanced techniques such as LC–MS/MS to identify specific binding sites on proteins and examine how different polyphenol structures affect binding efficiency and complex functionality. It should also clarify how polyphenol autoxidation and its by-products influence protein stability and function. Further, future work should optimize processing parameters such as pH, temperature, and time, and technological factors such as ultrasound power and HHP pressure, to increase the desired properties of protein-polyphenol complexes, such as encapsulation efficiency and antioxidant activity. Most investigations examine protein-polyphenol complexes in purified systems, whereas real food matrices can substantially alter their behavior. Studies should therefore evaluate multicomponent, lipid-rich products and identify the roles of individual protein fractions in complex sources such as insect proteins. Long-term safety and stability remain poorly understood, particularly regarding polyphenol depletion and protein–quinone interactions during storage. Limited scale-up data, insufficient modeling, and weak analytical verification continue to restrict industrial implementation.

Most studies have examined only a limited set of proteins, such as soy protein isolate, whey protein isolate, and ovalbumin, and polyphenols, such as catechin and EGCG. Expanding the scope with a wider range of plant and animal proteins, as well as more diverse polyphenols, will reveal new interactions and functionalities. Comparative analyses of diverse protein sources would enable the identification of the most suitable candidates to improve polyphenol bioactivity and bioavailability. Finally, ternary systems, such as protein–polysaccharide–polyphenol complexes, provide enhanced functionality and stability in comparison to binary systems. Future investigations should examine these multi-component systems to develop sophisticated delivery platforms for bioactive compounds.

## 10. Conclusions

We collected, summarized, and discussed recent advancements that support the application of protein-polyphenol complexes across the fields of food science, pharmaceuticals, and materials engineering. The molecular mechanisms of these conjugations, both covalent and non-covalent, that govern the formation of these complexes, are explained in detail. The review emphasizes that various processing technologies, including emerging non-thermal methods such as cold plasma and ultrasound, can be precisely controlled to engineer complexes with specific characteristics. These technologies offer sustainable and efficient pathways to create novel functional ingredients, moving beyond the limitations of traditional chemical or thermal treatments. Moreover, it is important to emphasize the understanding of the mechanisms by which protein-polyphenol complexes alter the structural and functional properties of the constituent molecules. These synergistic bioactivities promote potential benefits for human health due to their exceptional antioxidant properties among single plant-based resources. Additionally, improvement in reducing oxidative stress, bioavailability, and neuroprotection leads to managing type 2 diabetes and disease prevalence. Further, the formation of these complexes is not only a simple additive process but also contains unique characteristics that hold significant therapeutic and functional value. From an applied perspective, protein-polyphenol complexes can be employed to develop active packaging films, stabilize complex emulsions, enhance the functionality of plant-based proteins, and optimize inks for food 3D printing as well. These applications address growing consumer demands for sustainable, healthy, and highly functional products. Hence, there is a need for many studies to translate fundamental molecular knowledge into practical, high-impact applications and to contribute to achieving the objectives of the circular economy and the United Nations’ Sustainable Development Goals (SDGs).

## Figures and Tables

**Table 1:** Summary of reaction conditions, secondary structure change, tertiary structure change, and functional effects of protein-polyphenol complexes.

Protein	Polyphenol	Reaction Condition	Secondary Structure Change	Tertiary Structure Change	Functional Effects *	References
**Covalent bonding**						
Myofibrillar Protein (MP) from Grass Carp	Tea Polyphenols (TP)	Hydroxyl radical oxidation (Fe^3+^/ascorbic acid/H_2_O_2_); 3 h at 37 °C, then 12 h at 4 °C.	Oxidation reduced α-helix content and increased β-sheet/β-turn structures. Low TP concentrations inhibited α-helix loss. High TP concentrations caused denaturation.	MP surface hydrophobicity decreased at all TP levels. High TP concentrations induced irregular protein aggregation.	Low TP concentrations inhibited protein oxidation. ↑ Gel properties. Excessive TP exhibited pro-oxidative effects, reducing stability and gel capacity.	[3]
Zein	Ferulic Acid (FA)	pH 3.0, 5.0, 7.0, 9.0.	Not reported	Surface hydrophobicity decreased. Tyrosine residue exposure increased.	FA binding significantly enhanced antioxidant activity at all pH levels, with maximum activity at pH 7.0.	[4]
Soy protein isolate	(−)-Epigallocatechin gallate (EGCG)	Alkali treatment (pH 9.0). Free radical mediation. Enzymatic method. All with ultrasound assistance (450 W, 15 min).	EGCG grafting reduced α-helix content and increased β-sheet structures. Ultrasound assistance further decreased α-helices.	Hydrophobic group exposure increased microenvironment polarity. Fluorescence intensity decreased with blue shift (quenching effect).	Covalent bond formation (C–N and C–S). Ultrasound-assisted alkali treatment achieved the highest grafting efficiency. ↑ Emulsification, antioxidant activity, and α-glucosidase inhibition.	[5]
Pea protein isolate (ternary conjugate with maltodextrin)	Epigallocatechin-3-gallate (EGCG)	Glycation (PPI-MD). Covalent reaction under alkaline conditions.	Combined glycation and covalent reaction reduced α-helix content. and increased β-sheet. Random coil structures.	Lowest surface hydrophobicity observed. EGCG introduction increased steric hindrance and blocked hydrophobic residues.	Highest emulsification and DPPH scavenging activity. Effective pH-controlled curcumin carrier with 92.3% loading efficiency.	[6]
Potato protein	Quercetin	Conjugation/complexation at pH 7.0	Interactions decreased α-helix content and slightly increased β-sheet content, indicating protein unfolding.	Not reported	↓ Free amino groups indicate potential covalent interactions. Formation of hybrid protein-quercetin particles for Pickering emulsion stabilization.	[44]
Soy protein fibrils (curcumin-loaded)	(−)-Epigallocatechin gallate	EGCG remodeling of curcumin-loaded protein fibrils to form hydrogels; heat treatment for gel network formation	Not reported	EGCG adsorption and deposition on the fibril surface via hydrophobic and electrostatic interactions.	Formation of stable, uniform gel network. ↑ Thermal and UV stability of co-encapsulated compounds. ↑ Curcumin anticancer activity.	[71]
Soy protein isolate (film)	Mangosteen peel extract	Film incorporation (1–15% MPE content).	MPE incorporation decreased β-sheet content and increased β-turn and random coil structures.	Binding via hydrogen bonds and hydrophobic interactions.	↑ Antioxidant and antibacterial film properties. ↑ UV-visible light barrier performance.	[74]
**Non-covalent bonding**						
Whey protein isolate in oil-in-water emulsions	Resveratrol	Addition to WPI-stabilized emulsions (0.2–2.0 mM); 10-day storage study	Not reported	Tryptophan residues experienced oxidative stress. Protein oxidation is localized primarily at the oil-water interface.	Resveratrol provided dose-dependent protection against protein oxidation. ↓ Carbonyl formation and N-formylkynurenine generation during storage.	[2]
Myofibrillar protein from silver carp (film)	Oxidized phenols (caffeic acid, p-coumaric acid, eriodictyol, naringenin)	Cross-linking via oxidation at pH 12.0; heat treatment (80 °C, 30 min); LDH addition for nanocomposite films.	Dose-dependent protein unfolding with α-helix conversion to random coils Oxidized caffeic acid and eriodictyol caused the greatest structural degradation.	Oxidized polyphenols alone reduced hydrophobic accessibility. LDH combination increased surface hydrophobicity.	Covalent binding (C-S/C-N bonds) reduced sulfhydryl and amino groups. ↑ Mechanical properties (tensile strength up to 17.4 MPa). ↑ Gas and water barrier performance.	[17]
Plant/animal proteins (SPI, WPI, CP, TBP)	Apple polyphenols (AP)	Non-covalent binding (24 h, 37 °C, pH 7.0)	Interactions reduced α-helix proportion and increased β-sheet content of all proteins.	↓ Protein surface hydrophobicity. ↓ Fluorescence intensity (quenching effect). Binding via hydrogen bonds and hydrophobic interactions.	↑ Antioxidant capacity (highest in TBP-AP). ↑ Carbohydrate-hydrolyzing enzyme inhibition. ↑ AP bio-accessibility (TBP-AP: 74.01%) through gastric protection-intestinal release mechanism.	[29]
Egg white lysozyme	Theaflavin	Non-covalent binding in PBS (pH 7.4).	Theaflavin binding altered lysozyme secondary structure.	Fluorescence quenching occurred. ↓ Surface hydrophobicity. Binding primarily via electrostatic forces.	Binding sites overlapped with allergenic epitopes, potentially reducing egg allergenicity. ↓ Theaflavin antioxidant activity.	[30]
β-Casein	Coffee flavonoids (apigenin, luteolin, quercetin, EGCG)	Non-covalent interactions	Interactions increased random coil structure and decreased α-helix proportion, creating a more open protein conformation.	↓ Surface hydrophobicity. ↓ Fluorescence intensity (quenching effect). Binding mechanisms: luteolin/quercetin via hydrogen bonds and van der Waals forces; apigenin/EGCG via hydrophobic interactions	Superior antioxidant activity through synergistic action. ↑ Solubility, emulsifying activity, and foaming ability. Reduced emulsifying stability. EGCG demonstrated the strongest binding affinity.	[43]
Goat lactoferrin	Pterostilbene	Complexation with optimal binding at PTE > 60 μM/g protein; UV stability assessment.	Complexation decreased β-sheet content and increased β-turn structure.	Fluorescence quenching with blue shift (323 → 314 nm). ↑ Surface hydrophobicity.	↑ Foaming and emulsifying activities. Maintained PTE antioxidant capacity under UV irradiation. ↑ PTE bioaccessibility.	[72]
**Both covalent and non-covalent bonding**						
β-Lactoglobulin	Chlorogenic acid (CA)	Complex formation with/without ultrasound pretreatment (25 kHz, 900 W, 20 °C, 2 h).	Ultrasound treatment altered secondary structure. CA binding promoted spatial structure modifications.	Ultrasound reduced particle size to <50 nm. CA bound via hydrophobic interactions in internal cavity.	↑ CA binding capacity. Complex formation enhanced antioxidant activity. Enabled curcumin encapsulation.	[7]
Soy protein isolate	Catechin	Non-covalent binding (pH 7.0, oxygen-free) versus covalent binding (pH 9.0, oxygen-rich).	Protein-catechin interactions disrupted secondary structure organization.	↑ Hydrophobic residue exposure to hydrophilic environment. Strong fluorescence quenching at high catechin concentrations.	Covalent complexes demonstrated superior stability. ↑ Catechin binding capacity (maximum 132.09 nmol/mg protein). ↑ Antioxidant activity and catechin bioaccessibility (up to 86%).	[12]
Soy protein isolate (SPI)	Epigallocatechin gallate (EGCG)	Covalent vs. non-covalent modification Simulated gastrointestinal digestion.	Conformational changes occurred (inferred from anthocyanin analogy studies).	Not reported	Covalent attachment enhanced EGCG protection during digestion (95.30% retention). ↓ Protein digestibility compared to native SPI.	[36]
Whey protein concentrate	Quercetin	Comparative study: non-covalent (neutral pH) vs. covalent (alkaline pH) interactions.	Interactions decreased β-sheet content and increased random coil structure, causing secondary structure unfolding.	Fluorescence quenching with a red-shifted peak. ↓ Surface hydrophobicity.	Covalent complexes: 4.75× more binding sites and higher quercetin capacity. ↑ Foaming/emulsifying activities and antioxidant capability. Non-covalent binding reduced solubility via aggregation.	[70]

* ↑ and ↓ indicate an increase and decrease in the corresponding properties, respectively.

**Table 2:** Synergistic bioactivities, quantitative results, and underlying mechanisms of protein-polyphenol complexes.

Protein Type	Polyphenol	Interaction Type	Synergistic Mechanism	Key Quantitative Results	References
**Antioxidant activity**					
Pea protein isolate (PPI)	EGCG (with maltodextrin)	Ternary Complex (Maillard-induced glycation & Covalent)	Maillard-assisted protein unfolding increases exposure of reactive –OH groups.	DPPH scavenging reaches 87.1%, representing a 2.4-fold enhancement compared with native PPI.	[6]
Soy Protein Isolate (SPI)	EGCG (with Anionic Polysaccharides)	Covalent (SPI-EGCG) & Non-covalent (Polysaccharide)	Polysaccharide shell provides surface protection while preserving free phenolic –OH activity.	DPPH scavenging capacity of ternary complexes remains consistently above 70%	[9]
Soy Protein Isolate (SPI)	Catechin	Covalent & non-covalent	Covalent cross-linking forms a compact matrix that shields catechin from oxidative stress (light, O_2_, pH).	Storage Stability: Catechin residue ratio significantly higher than free catechin (after 7 days at 20 °C). Bioavailability: Increased to ~86% (vs. 30% catechin free).	[12]
Tartary Buckwheat (TBP), Whey (WPI), Chickpea (CP), Soy (SPI).	Apple polyphenols (AP).	Non-covalent (H-bonds, hydrophobic, electrostatic).	Proteins shield AP from gastric acid and release antioxidant peptides during digestion.	Radical Scavenging (DPPH): SPI: Increased 4.64% → 89.48%. WPI: Increased 13.23% → 90.72%.	[29]
**Enzyme inhibition & metabolic**					
Tartary Buckwheat (TBP), Whey (WPI), Chickpea (CP), Soy (SPI).	Apple Polyphenols (AP).	Non-covalent (H-bonds, hydrophobic, electrostatic).	Enzyme inhibition and intracellular redox regulation via restoration of endogenous antioxidant systems (SOD, CAT, GSH-Px).	Enzyme inhibition: WPI–AP exhibited the strongest inhibition of α-amylase (55.73%) and α-glucosidase (43.49%), comparable to acarbose. Cellular protection (WPI–AP): GSH-Px activity restored to 122.31 U/mg; lipid peroxidation markedly reduced (0.072 → 0.009 nmol/mg).	[29]
Insulin	Hydroxytyrosol	Tyrosyl-region binding (non-covalent)	Maintains insulin in active/non-toxic form → Prevents amyloid-associated diseases.	Completely inhibited insulin amyloid aggregation and reversed amyloid-induced cytotoxicity.	[104]
Nano-curcumin (Cur-ASP)	Curcumin	Encapsulation/Surface binding	The massive surface area of nanoparticles exposes more functional groups to block enzyme binding sites more effectively than free curcumin.	α-Amylase: 68.67% inhibition (≈Acarbose 68.74%). α-Glucosidase: 58.30% inhibition (IC_50_ 47.62 mg/mL).	[105]
**Antibacterial**					
Soy Protein Isolate (SPI)	Catechin	Covalent & non-covalent	Catechin combined with protein not only acts as a direct antibacterial but also stimulates the growth of beneficial gut bacteria.	Enhanced durability of catechin under gastrointestinal conditions. Controlled release of polyphenols during digestion.	[12]
Red Algae Extract (JRPE)	Kaempferol, Quercetin, Chlorogenic Acid	Multi-component Synergy	Inhibits Sortase A/B enzymes (essential for bacterial adhesion) and penetrates to damage bacterial DNA.	Inhibited 98.6% of *S. Pyogenes* and 99.8% of *E. Faecalis.* Outperformed Ampicillin and Amoxicillin.	[106]
**Bioavailability**					
Pea Protein (PPI) + Maltodextrin	Curcumin	Covalent (Ternary Conjugate)	The combination of glycation and covalent binding creates a thick interfacial layer that reduces curcumin exposure and provides slow release in the intestine.	Loading efficiency: 92.3%. Curcumin retention of 84% after 15 days at 25 °C, markedly higher than that in ethanol.	[6]
Soy Protein Isolate (SPI)	Catechin	Covalent (Irreversible C-N/C-S bonds)	Covalent cross-linking firmly anchors catechin to the protein matrix, limiting enzyme–complex dissociation and protecting the polyphenol from oxidative degradation.	Bioaccessibility: Enhanced to ~86%, compared with ~30% for free catechin.	[12]
Plant Proteins (TBP, WPI, CP, SPI)	Apple Polyphenols (AP)	Non-covalent (H-bonds, hydrophobic)	Protein carrier prevents early gastric degradation → Intestinal release.	TBP-AP reached 74.01% bio-accessibility, nearly double that of free AP (40.83%).	[29]
Serum Albumins (HSA, BSA)	EGCG, ECG, Catechin	Non-covalent (Ionic/H-bonding)	Transport and Solubility: Albumins serve as versatile carriers that transport lipophilic polyphenols in aqueous environments, increasing their bioactivity in milk-based systems.	Binding efficacy reached 40–65%; stability follows the order EGCG > ECG > C.	[58]
**Anticancer**					
Soy Protein Fibrils (UF)	Curcumin (Cur) & EGCG	Non-covalent (Hydrophobic and Electrostatic interactions)	EGCG adsorbs and deposits on the surface of the UF-Cur complex (UFC), remodeling it into a uniform supramolecular gel network that enhances stability and promotes cellular internalization.	Caco-2 cell viability reduced to 8.29% at 5 µg/mL Cur, compared with >95% for free polyphenols.	[71]

## Data Availability

No new data were created or analyzed in this study. Data sharing is not applicable to this article.
